# Photoinhibition of comammox reaction in *Nitrospira inopinata* in a dose- and wavelength-dependent manner

**DOI:** 10.3389/fmicb.2022.1022899

**Published:** 2022-12-15

**Authors:** Ekaterina Y. Gottshall, Bruce Godfrey, Bo Li, Britt Abrahamson, Wei Qin, Mari Winkler

**Affiliations:** ^1^Department of Civil and Environmental Engineering, University of Washington, Seattle, WA, United States; ^2^Department of Microbiology and Plant Biology, Institute for Environmental Genomics, University of Oklahoma, Norman, OK, United States

**Keywords:** comammox, nitrification, photoinhibition, light intensity, diurnal cycle

## Abstract

Apparent contribution of complete ammonia-oxidizing organisms (comammox) to the global nitrogen cycle highlights the necessity for understanding niche differentiation of comammox bacteria among other ammonia oxidizers. While the high affinity for ammonia of the comammox species *Nitrospira inopinata* suggests their niche partitioning is expected to be centered in oligotrophic environments, their absence in nutrient-depleted environments (such as the oceans) suggests that other (abiotic) factors might control their distribution and spatial localization within microbial communities. Many ammonia- and nitrite-oxidizing organisms are sensitive to light; however, the photosensitivity of comammox has not been explored. Since comammox bacteria encode enzymatic machinery homologous to canonical ammonia-and nitrite-oxidizers, we hypothesized that comammox *N. inopinata,* the only available pure culture of this group of microorganisms, may be inhibited by illumination in a similar manner. We evaluated the impact of light intensity, wavelength, and duration on the degree of photoinhibition for cultures of the comammox species *N. inopinata* and the soil ammonia-oxidizing archaea *Nitrososphaera viennensis*. Both species were highly sensitive to light. Interestingly, mimicking diurnal light exposure caused an uncoupling of ammonia and nitrite oxidation in *N. inopinata,* indicating nitrite oxidation might be more sensitive to light exposure than ammonia oxidation. It is likely that light influences comammox spatial distribution in natural environments such as surface fresh waters according to diurnal cycles, light attenuation coefficients, and the light penetration depths. Our findings therefore provide ecophysiological insights for further studies on comammox both in field and laboratory settings.

## Introduction

Biological nitrification results in ammonia oxidation to nitrate, which is performed in two steps: first, ammonia-oxidizing bacteria or archaea (AOB/AOA) oxidize ammonia to nitrite and second, nitrite-oxidizing bacteria (NOB) oxidize nitrite to nitrate (Könneke et al., 2005; Leininger et al., 2006). In 2015, a new nitrification player was identified to perform complete ammonia oxidation (comammox) to nitrate, *via* nitrite as an intermediate, within a single organism (Daims et al., 2015; van Kessel et al., 2015). Since then, lineage II *Nitrospira* comammox species have been widely detected in forest and agricultural soils, freshwater and brackish sediments, and waste and drinking water treatment plants (Pinto et al., 2016; Camejo et al., 2017; Pjevac et al., 2017; Fowler et al., 2018; Xia et al., 2018; Spasov et al., 2020; Yang et al., 2020). Comammox species were shown to be active participants and to even dominate some wastewater systems (Roots et al., 2019; He et al., 2022). Despite its widespread occurrence, major environmental variables governing the distribution and abundance of comammox and hence their role in microbial ecosystems are not well characterized.

Environmental distribution, abundance, physiology, and genetics studies revealed that the canonical ammonia-oxidizing microorganisms (AOM) AOB and AOA coexist in many ecosystems. AOA have been shown to outnumber AOB in diverse environments, including soils (Leininger et al., 2006), oceans (Wuchter et al., 2006), streams (Merbt et al., 2017) and alpine lakes (Auguet et al., 2012) highlighting their ecological significance and potential factors for niche differentiation between AOA and their bacterial counterpart AOB (Hatzenpichler Roland, 2012; Koch et al., 2019). While kinetic parameters such as high affinities for substrate influence AOM adaptation to their environmental stressors, abiotic factors likewise determine nitrification communities’ distribution and activity in ecosystems. Several studies of both AOB and AOA in pure cultures in laboratory experiments and in field studies demonstrated differential sensitivity to various abiotic factors including pH (Nicol et al., 2008; Sun et al., 2020), temperature (Tourna et al., 2008), ammonium concentration (Martens-Habbena et al., 2009; Verhamme et al., 2011), and light (Merbt et al., 2012) in both natural and man-made systems. These factors appear to control AOB, AOA, and NOB relative abundances and activities, suggesting distinct physiological adaptations for each group.

Comammox species showed comparably high affinity for ammonia (K_m(app)(NH3)_ ≈ 63 nM) as some AOA species, suggesting their adaptation to low ammonia habitats (Kits et al., 2017; Jung et al., 2022). Indeed, similarly to AOA (Leininger et al., 2006; Wuchter et al., 2006; Merbt et al., 2012; Auguet and Casamayor, 2013), comammox have been found in ammonia-depleted fresh water and engineered systems (Bartelme et al., 2017; Hu and He, 2017; Pjevac et al., 2017; Fowler et al., 2018; Orellana et al., 2018). Unlike open oceans which are often dominated by AOA, other geographical areas such as estuaries (Bernhard and Bollmann, 2010) and salt marshes (Wang et al., 2021b) have more complex ecological relationships and diverse AOM community compositions. Specifically, comammox was found to be more abundant than AOB in salt marshes (Wang et al., 2021b), coastal waters and sediments (Xia et al., 2018), and river sediments (Zhao et al., 2020). Only a few studies focused on the abiotic factors influencing comammox distribution. Salinity was shown to have a negative correlation with comammox abundance (Sun et al., 2020), which was earlier predicted to be absent (Daims et al., 2015) and thus far was not detected in the marine environments. Some studies demonstrated higher temperatures to be beneficial for comammox growth (Fowler et al., 2018; How et al., 2019), while others found a correlation with lower temperatures and comammox abundance (Gonzalez-Martinez et al., 2016). It is clear, however, that comammox is an important player in many ecosystems, which suggests the importance of further evaluation of key environmental variables controlling comammox spatial distribution within its natural habitats.

Light inactivation of ammonia monooxygenase (AMO) in AOB has been known for several decades, requiring *de novo* synthesis of the enzyme to resume microbial activity (Schoen and Engel, 1962; Hyman and Arp, 1992). Recent studies of AOA pure cultures revealed similar trend in the photoinhibition of their ammonia oxidation activities (French et al., 2012; Merbt et al., 2012; Qin et al., 2014, 2017) although the biochemical mechanisms associated with photoinhibition require more research. To date, there are no reports of light sensitivity of comammox. We hypothesized that comammox may be sensitive to light as it possesses AMO enzymatic machinery and other enzymes that were shown to be light-sensitive in other AOM species. Environmental factors create a complex interplay generating various patterns of nitrifying organisms in ecosystems. To better understand the key physicochemical parameters that exert major controls on the spatial variability and dynamics among AOA, AOB, and comammox, it is necessary to study the influence of as many abiotic drivers as possible including light. The aims of this study were to understand how different light intensities, wavelengths, and duration of irradiation influence ammonia (ammonia represents total ammonia throughout the manuscript unless otherwise mentioned, NH_3_ + NH_4_^+^) oxidation by the comammox species *Nitrospira inopinata* and soil AOA species *Nitrososphaera viennensis* and to help explain the differential distribution and activity of these AOM species in terrestrial and aquatic systems.

## Materials and methods

### Microorganisms and growth conditions

*Nitrospira inopinata* (comammox) was incubated in a limited mineral media (composition per little: 1 g KH_2_PO_4_; 1.5 g KCl; 1 g MgSO_4_**∙**7H_2_O; 11.68 g NaCl; 4 g CaCO_3_, 1 ml trace element solution (TES), and 1 ml of selenium-wolfram solution (SWS)) aerobically at 37°C pH 7.5 in the presence of 1 mM NH_4_^+^ in the dark without shaking (Daims et al., 2015). TES contained following (per liter): 34.4 mg MnSO_4_**∙**1H_2_O; 50 mg H_3_BO_3_; 70 mg ZnCl_2_; 72.6 mg Na_2_MoO_4_**∙**2H_2_O; 20 mg CuCl_2_**∙**2H_2_O; 24 mg NiCl_2_ **∙** 6H_2_O; 80 mg CoCl_2_**∙**6H_2_O; and 1 g FeSO_4_ **∙**7H_2_O. All salts except FeSO_4_**∙**7H_2_O were dissolved in 997.5 ml Milli-Q water and 2.5 ml of 37% HCl was added before dissolving the FeSO_4_ **∙** 7H_2_O salt. SWS contained (per liter): 0.5 g NaOH; 3 mg Na_2_SeO_3_**∙**5H_2_O; and 4 mg Na_2_WO_4_**∙**2H_2_O (Daims et al., 2015). Ammonia-oxidizing soil archaea *Nitrososphaera viennensis EN76* strain was also incubated in AOA medium for maintenance (Bollmann et al., 2011) or in CaCO_3_ containing mineral media for the photoinhibition experiments at 30°C pH 7.5 in the presence of 1 mM NH_4_^+^ in the dark without shaking supplemented with 1 mM sodium pyruvate. Incubations were inoculated with a 10% inoculum from late exponential phase maintenance cultures. The *N. inopinata* and *N. viennensis* maintenance cultures demonstrate a constant ammonia oxidation rate in the late exponential phase and have a cell density on the order of 10^7^ cells per mL culture. All cultures were incubated until either ammonia depletion or the nitrification rate approaches zero.

### Metabolic activity measurements

Metabolic activity of microbial strains was monitored through regular measurements of ammonium (NH_4_^+^), nitrite (NO_2_^−^), and total oxidized nitrogen (TON; NO_2_^−^ + NO_3_^−^) concentrations in media during incubation. NH_4_^+^, NO_2_^−^, and NO_3_^−^ were measured using a colorimetric method with GalleryTM Automated Photometric Analyzer (Thermo Fisher Scientific, Waltham, MA U.S.A.) with the total oxidized nitrogen (TON)-Nitrite and ammonia reagents calibrated using sodium nitrite (NaNO_2_) and ammonium chloride (NH_4_Cl) standards. Nitrate (NO_3_^−^) concentrations were determined by subtracting nitrite from the TON concentrations. Samples were processed immediately after collection.

### Illumination settings and apparatus

**Figure 1:**
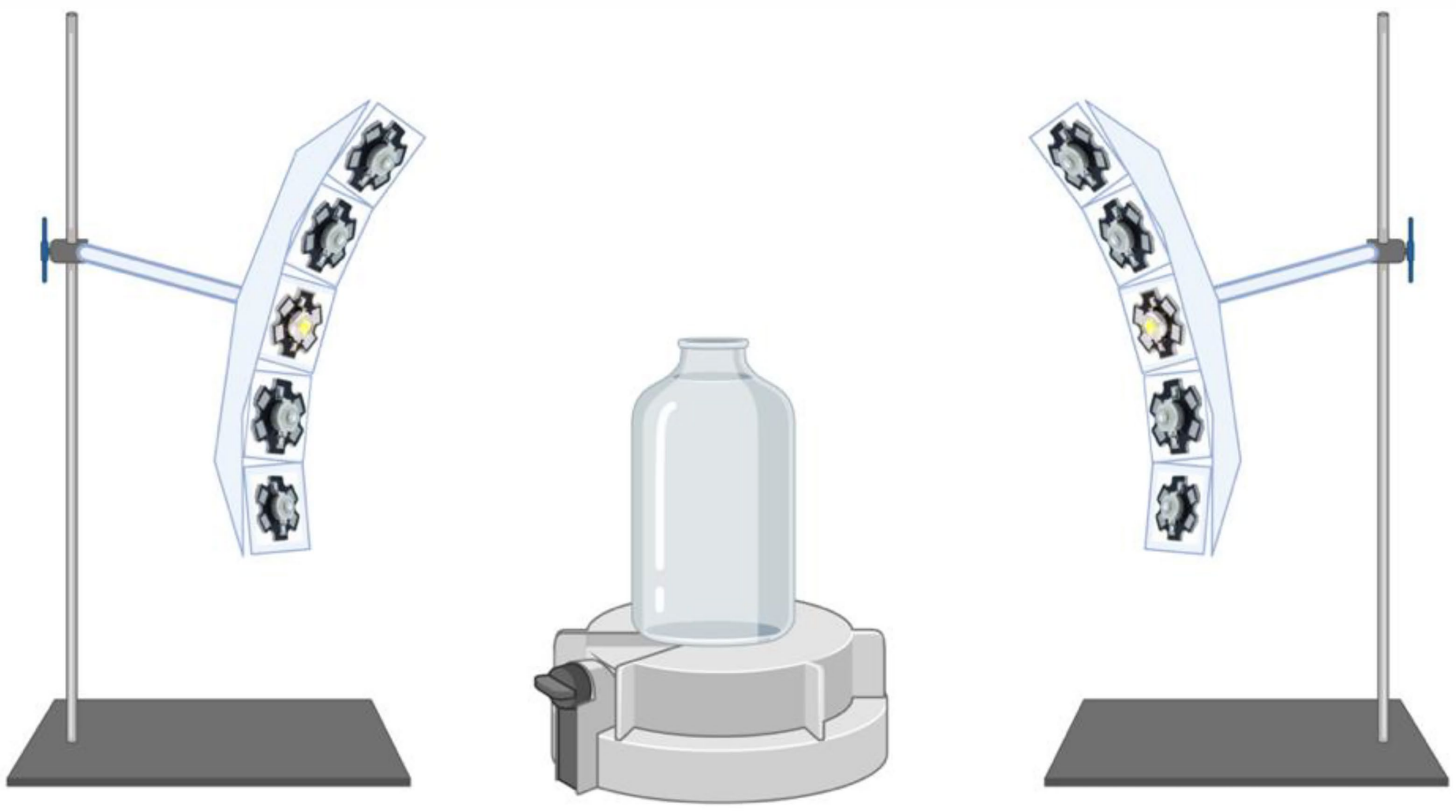
Schematic of the illumination apparatus. Cool LED polychromatic light sources are shown to be directed toward the glass bottle *via* collimating lens to focus the emitted light down to a 5 degree beam; Light meter (Apogee Instruments) located at the bottom of the glass bottle is displayed.

Illumination was provided from a cool LED polychromatic light source. The duration and intensity of visible range light wavelengths (400–680 nm) in the glass culturing bottle was controlled using a pyranometer-style light meter which integrates radiation received over 180 degrees, placed inside the bottles (Apogee Instruments (Logan, Utah) SP-420 Smart Pyranometer with ApogeeConnect software; Spectral response 400 nm to 1,100 nm over 180 degrees). The LED light source was assembled using 2 curved aluminum plates each carrying 8 individual nominally 3 W LED’s on “star” heatsinks. Five types of LEDs were used to cover the full white light spectrum from 400 nm to 680 nm. Each LED was equipped with a collimating lens to focus the emitted light down to a 5° beam and arranged so that the light spots were all concentrically focused on the test bottles (Figure 1). Some of the LEDs were covered with foil to produce the blue-green spectrum or the yellow-red spectrum illumination regimes.

Experiments were carried out at 37°C for *N. inopinata* and at 30° C for *N. viennensis*. Although 30°C is not the optimal growth temperature of *N. viennensis,* a culturing temperature of 30°C was selected to compare the ammonia oxidation rate of *N. viennensis* across studies (Straka et al., 2019). *Nitrospira inopinata* and *N. viennensis* cultures were exposed to light intensities selected to reflect naturally occurring conditions during a clear summer day in open (ocean-type upper water column) areas (500 and 800 μE m^−2^ s^−1^) and the lower intensities (60 and 15 μE m^−2^ s^−1^) simulating conditions in shaded (e.g., lower oceanic column) areas for various duration regimens. Control cultures were incubated in the dark in the same incubator. All cultures were stirred to ensure uniformity of light exposure and grown in triplicates. Light intensities, duration of illumination, and wavelengths of irradiation are indicated for each experiment in the results sections and in corresponding figures.

Experiments were carried out at specific light intensities, wavelengths, and exposure durations. In all photoinhibition experiments, *N. inopinata* and *N. viennensis* cultures were supplemented with 1 mM ammonia and exposed to specific light intensities (15, 60, 500, and/or 800 μE m^−2^ s^−1^) for either 15 min (both) or 60 min (*N. inopinata* only). Immediately after light exposure, nitrogen metabolite (NH_4_^+^, NO_2_^−^, and NO_3_^−^) concentrations were measured, and the cultures were placed in the dark. Nitrogen metabolites were monitored until either ammonia depletion or the absence of nitrification activity for at least 14 days. The nitrification rate was calculated as the rate (natural logarithm transformed nitrogen species) of TON (NO_2_^−^ + NO_3_^−^) and NO_2_^−^ accumulation for *N. inopinata* and *N. viennensis, respectively,* as in previous studies (Qin et al., 2014). Photoinhibition was calculated as percent decrease in the exponential nitrification rate after light exposure compared to the dark control (Qin et al., 2014). The response of *N. inopinata* to specific wavelengths was evaluated by exposing cultures to 60 μE m^−2^ s^−1^ light with wavelengths of either 400–550 nm (blue-green spectrum) or 550–680 nm (red-yellow spectrum) for 15 min. Nitrogen metabolite concentrations were monitored until ammonia depletion. The experiments evaluating the impact of hydrogen peroxide scavengers on *N. inopinata* were conducted in the manner described above for the photoinhibition experiments, except cultures were supplemented with 1 mM of sodium pyruvate prior to light exposure.

For continuous illumination experiments, changes in nitrogen concentrations were assessed for 4 days before the light exposure started, test set of cultures was then placed under continuous light (15 μE m^−2^ s^−1^), while control set of cultures was kept in the dark. Nitrogen metabolites were monitored for 2 weeks or until all ammonia depletion (control). For diurnal experiments, initial nitrogen metabolite concentrations were measured prior to the initial light exposure. Nitrogen metabolites were monitored immediately after light exposure ended and before the next round of illumination begin (6 h light – 18 h dark recovery). Nitrite or nitrate accumulation in control and illuminated cultures were compared using the Student’s *t*-test (two-sample assuming unequal variances).

## Results

### Comammox *Nitrospira inopinata* is sensitive to light in a dose-and wavelength-dependent manner

**Figure 2:**
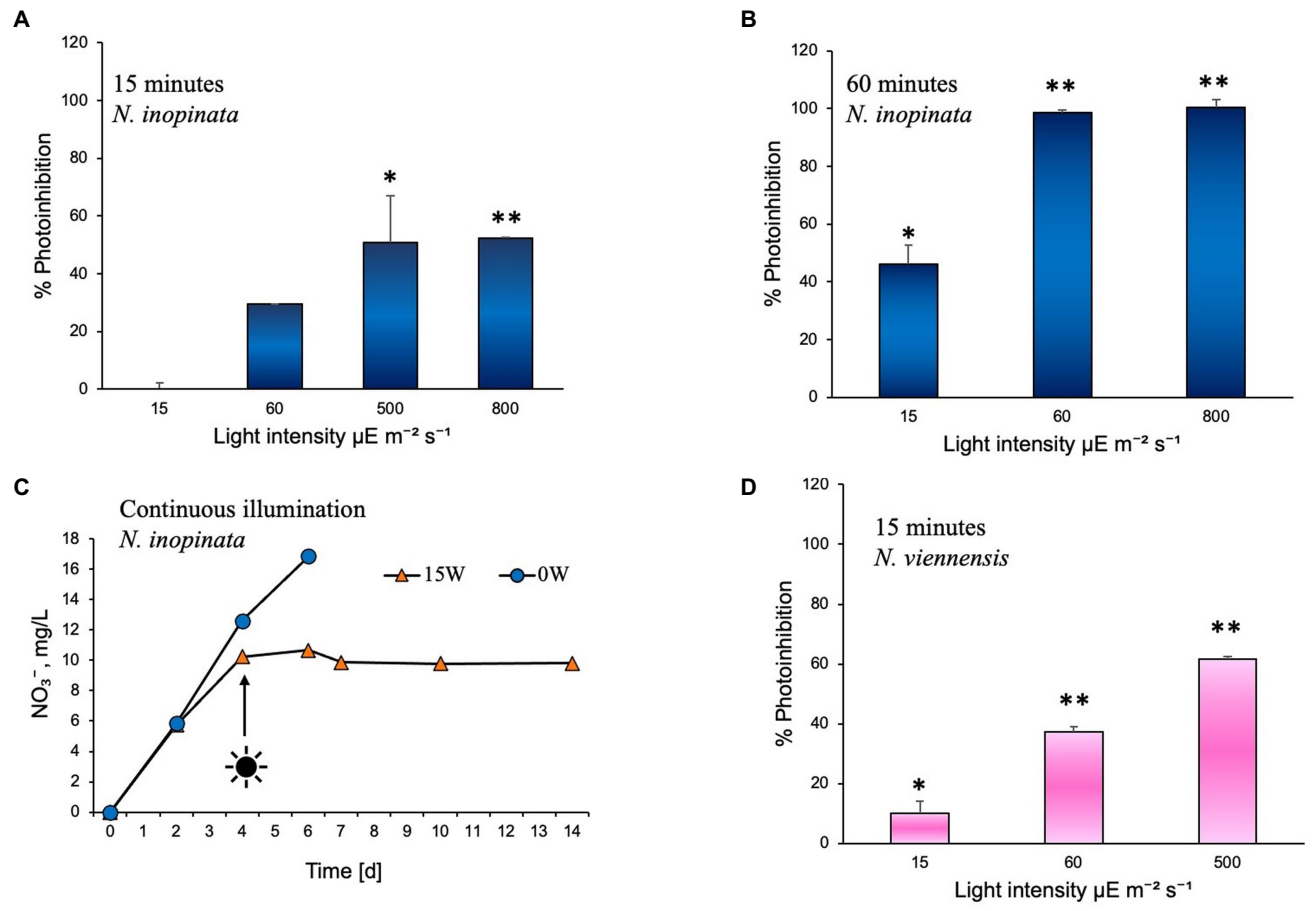
Photoinhibition of nitrification activity in *Nitrospira inopinata* represented as percent reduction of the rate of TON accumulation, compared to the dark control cultures incubated in parallel, after exposure to **(A)** 15, 60, 500, and 800 μE m^−2^ s^−1^ intensities for 15 min, **(B)** 15, 60, 500, and 800 μE m^−2^ s^−1^ intensities for 60 min. **(C)** Nitrate concentration vs. time during incubation under continuous illumination at 15 μE m^−2^ s^−1^ intensity. **(D)** Photoinhibition of nitrification activity in *Nitrososphaera viennensis* represented as percent reduction of the rate of TON accumulation, compared to the dark control cultures incubated in parallel, after exposure to 15, 60, and 500 μE m^−2^ s^−1^ intensities for 15 min. Data are presented as the mean and standard error of triplicate cultures. Error bars are smaller than symbol size in panel C. Significant differences between control and illuminated cultures are represented as *p < 0.05 and **p < 0.001.

The effect of white light on comammox *N. inopinata* nitrification activity was evaluated through controlled exposure to a polychromatic light. Nitrification activity was measured at various illumination intensities and intervals. Illumination intensities were selected to simulate exposure to dim light (15 μE m^−2^ s^−1^), shade light (60 μE m^−2^ s^−1^), bright light (500 μE m^−2^ s^−1^), and direct sunlight (800 μE m^−2^ s^−1^) in an aquatic environment. Cultures were exposed to these intensities under 15 to 60 min light followed by dark recovery phase, continuous light, and continuous dark regimens. For 15- and 60-min illumination experiments, cultures were exposed to light once; nitrification activity was then measured from the end of light exposure until all ammonia was either oxidized or no nitrification activity was detected, which defined the recovery period for each light treatment. Photoinhibition was therefore determined as percent decrease in the exponential nitrification rate after light exposure compared to the dark control (Figure 2 A, B). All cultures that were exposed to light experienced slower nitrification activity recovery in an intensity- and duration-dependent manner. After 15 min of exposure, *N. inopinata* was found to be increasingly inhibited by the light with elevated light intensities (Figure 2A). Cultures exposed to bright or direct sunlight (500 and 800 μE m^−2^ s^−1^) for 15 min demonstrated over 50% photoinhibition (Figure 2A; Supplementary Figures S1A–C). Lower intensities exposures for 15 min caused less photoinhibition; however, even shade light (60 μE m^−2^ s^−1^) resulted in substantial photoinhibition (Figure 2A; Supplementary Figures S1A–C).

Fifteen minutes of light exposure at 15 μE m^−2^ s^−1^ and 60 μE m^−2^ s^−1^ had no significant effect on ammonia (Supplementary Figure S1A) or nitrite oxidation (Supplementary Figure S1B) during the first 3 days of dark recovery; however, after 3 days, a delay in nitrification activity was observed (Supplementary Figures S1A–C). Fifteen minutes exposure to the 500 μE m^−2^ s^−1^ intensity light significantly slowed ammonia oxidation starting from the time cultures were placed in the dark (Supplementary Figure S1A) and slowed nitrite oxidation from around day two of dark recovery (Supplementary Figures S1B,C). Exposure to 800 μE m^−2^ s^−1^ light for 15 min blocked ammonium oxidation for 5 days, after which a slow recovery of activity took place (Supplementary Figures S1A–C).

Exposure to 15 μE m^−2^ s^−1^ light for 60 min slightly slowed ammonia oxidation for 2 days, after which the ammonia oxidation rate recovered (Supplementary Figure S1D). However, nitrite oxidation was more strongly affected by the 15 μE m^−2^ s^−1^ 60-min light exposure, with no nitrate production for the first 2 days followed by a slow recovery of nitrate accumulation over the next 5 days (Supplementary Figure S1F). Sixty-minute light exposure at 60 μE m^−2^ s^−1^ and 800 μE m^−2^ s^−1^ caused ammonia oxidation to completely cease (Supplementary Figures S1D–F). This reveals a significant difference in light sensitivity of the two steps in complete ammonia oxidation metabolism, with nitrite oxidation apparently being more sensitive to light exposure than ammonia oxidation.

In summary, the longer exposures (60 min) to light at 60 μE m^−2^ s^−1^ and 800 μE m^−2^ s^−1^ completely inhibited the nitrification activity of *N. inopinata*, which did not recover after over 2 weeks of dark incubation (Figure 2B; Supplementary Figures S1D–F), demonstrating high photosensitivity of comammox and its adaptation to very dim light intensities. Higher intensities or longer duration of illumination also caused a delay in resuming nitrification activity. Fifteen minute dim light exposure (15 μE m^−2^ s^−1^) lengthened nitrification activity only by approximately 24 h, while high light intensities (500 and 800 μE m^−2^ s^−1^) caused slower nitrification activity and a delay of 48–120 h in resuming the activity. Sixty minutes of dim light exposure caused a delay (48 h) and slower ammonia oxidation (Supplementary Figure S1).

To test the effect of continuous illumination on ammonia oxidation of *N. inopinata,* cultures were incubated in the dark first and after nitrification activity was established (day 4) flasks were set under continuous illumination. Continuous illumination at dim light intensity (15 μE m^−2^ s^−1^) completely ceased nitrification activity of *N. inopinata* (Figure 2C; Supplementary Figure S2), suggesting that nitrification activity is being performed only during the dark period.

Light inhibitory effect was also evaluated for soil AOA *Nitrososphaera viennensis* that demonstrated slower growth (measured as nitrite accumulation over time) relative to cultures grown in the dark. *N. viennensis* cultures were exposed to light once for 15 min at 15, 60, and 500 μE m^−2^ s^−1^ illumination intensities following dark recovery. Nitrogen concentrations and photoinhibition assessments were performed as for *N. inopinata* experiments described above and in Figure 2A. *N. viennensis* nitrification activity appears to be more disturbed across different light intensities than *N. inopinata* during the same illumination regimen (Figure 2D): approximately 10% more photoinhibition was observed under moderate to high (60–500 μE m^−2^ s^−1^) light intensities than for *N. inopinata.*

**Figure 3:**
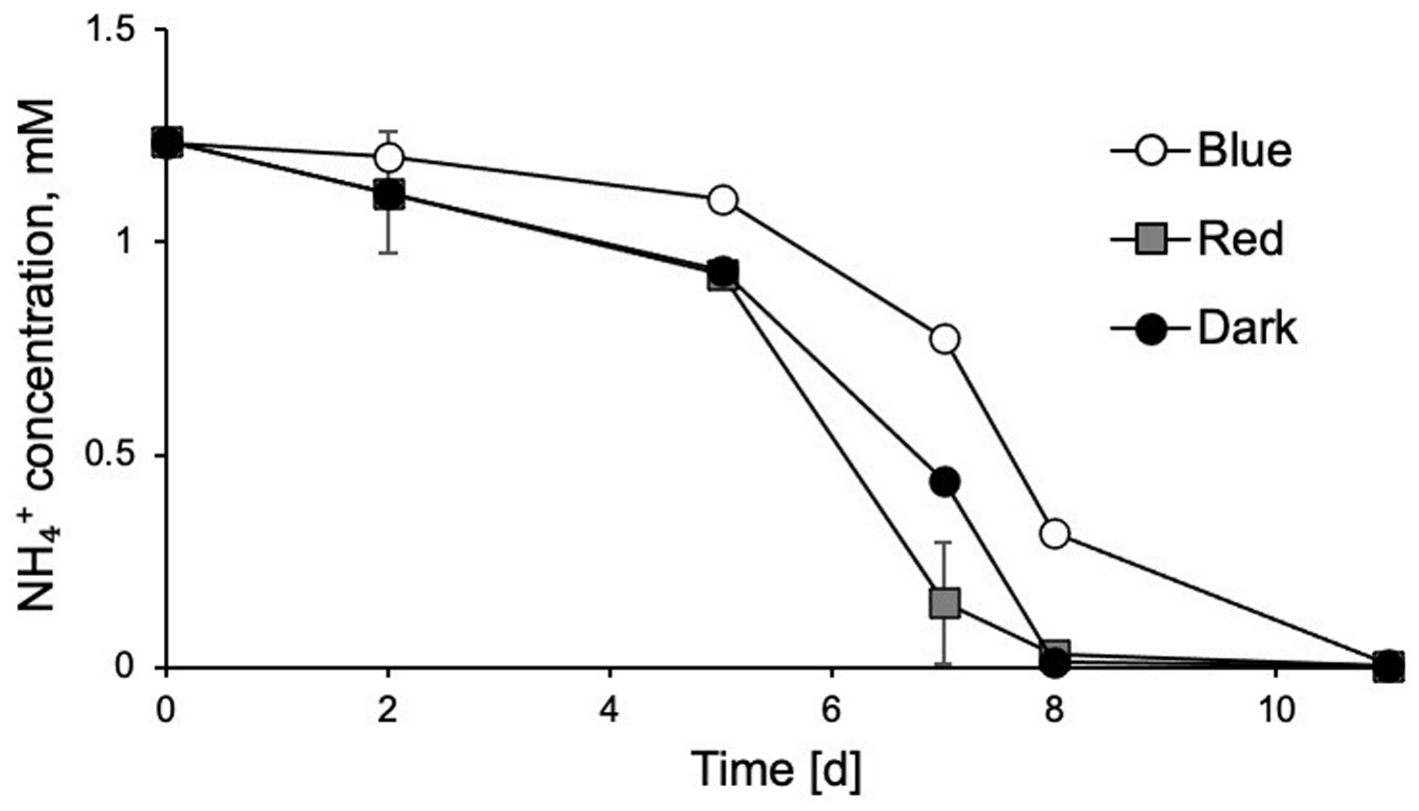
Exposure of *N. inopinata* to wavelengths of blue-green spectrum (450–550 nm) caused delayed ammonia oxidation in comparison to yellow-red spectrum (550–680 nm) wavelengths and dark control. Data are presented as the mean and standard error of triplicate cultures.

To further investigate which wavelength of light had the stronger inhibitory effects on *N. inopinata* nitrification activity, cultures were incubated at an illumination intensity of 60 μE m^−2^ s^−1^ lasting 15 min with blue/green (400–550 nm) and yellow/red (550–680 nm) lights. The light wavelength was controlled by adjusting wavelength range of a polychromatic light. Exposure to light with wavelengths shorter than 550 nm caused nitrification activity delay in *N. inopinata* (Figure 3; Supplementary Figures S3A,B), while light with longer wavelengths (over 550 nm) did not seem to disturb ammonia oxidation activity of *N. inopinata* (Figure 3; Supplementary Figures S3A,B).

### Hydrogen peroxide scavenger demonstrates protective effect against photoinhibition in comammox *Nitrospira inopinata*

**Figure 4:**
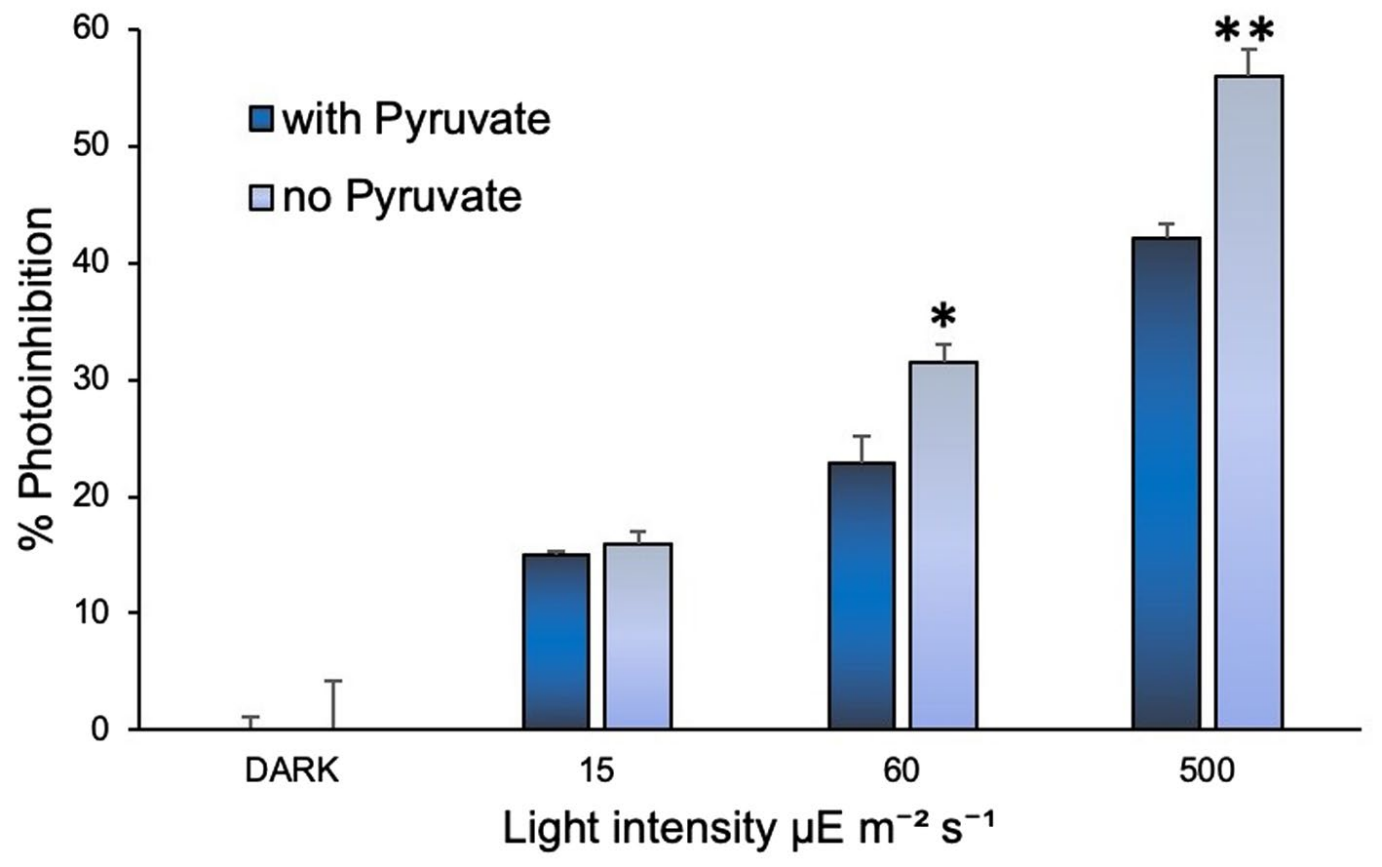
Sodium pyruvate supplementation positively affected *N. inopinata* growth when exposed to light at 60 and 500 μE m^−2^ s^−1^. Data are presented as the mean and standard error of triplicate cultures. Significant differences between control and illuminated cultures are represented as *p < 0.05 and **p < 0.001.

Addition of sodium pyruvate (1 mM) to the cultures of *N. inopinata* showed that it may play a protective role in its ammonia oxidation activity against photoinhibition (Figure 4). Significant effect was observed under regimens of over 60 and 500 μE m^−2^ s^−1^ irradiation (15 min light exposure), while cultures under dim light (15 μE m^−2^ s^−1^) did not seem to be affected by pyruvate amendment (Supplementary Figure S4). Pyruvate supplementation in *N. inopinata* dark control cultures had no discernable effects on their nitrification activity relative to pyruvate-free cultures (Supplementary Figure S4). *N. viennensis* was not evaluated for the pyruvate photoprotective effect since pyruvate significantly accelerates its growth rate in the dark (Tourna et al., 2011) and is part of its standard media composition.

### Diurnal cycle photoinhibition: Comammox *Nitrospira inopinata* second nitrification step is ceased

**Figure 5:**
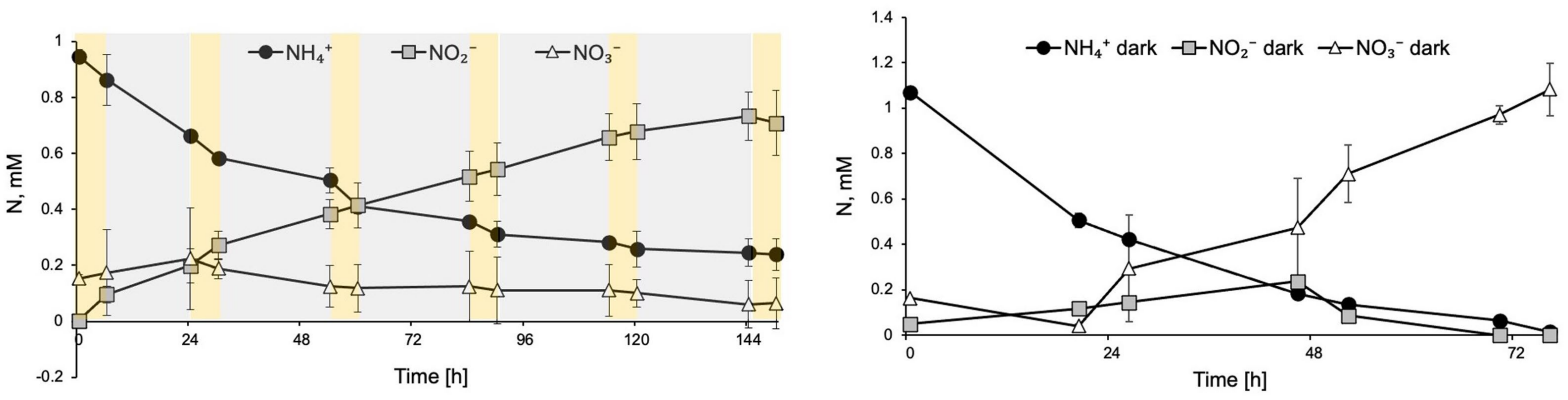
Diurnal light exposure of *N. inopinata* at 6 h light/18 h dark regimen caused ammonia oxidation delay, nitrite accumulation, and interruption of nitrate production. **(A)** Nitrogen concentration for N-NH_4_^+^, N-NO_2_^−^, and N-NO_3_^−^ are shown for diurnal *N. inopinata* exposure; **(B)** Nitrogen concentration for N-NH_4_^+^, N-NO_2_^−^, and N-NO_3_^−^ for *N. inopinata* cultures kept in the dark. Data are presented as the mean and standard error of triplicate cultures.

To evaluate the effect of diurnal cycles on *N. inopinata,* cultures were cyclically exposed to 6 h full spectrum visible light at 15 μE m^−2^ s^−1^ followed by 18 h dark recovery. Nitrification activity was measured during this regimen (Figure 5A). Interestingly, during light/dark cycles, *N. inopinata* failed to produce nitrate and instead, we detected nitrite accumulation. Ammonia oxidation rate was also reduced compared to the dark control (Figure 5B). This inhibition eventually resulted in a plateau of ammonia concentration at around 0.3 mM (Figure 5A) rather than complete ammonia oxidation. During first 24 h, nitrate concentration was slightly increasing; however later, it ceased to accumulate and even decreased.

## Discussion

The detrimental effect of light on ammonia monooxygenase (AMO) enzyme has been documented for many AOM (Lu et al., 2020a) and has been linked to the suppression of nitrification rates, variations in inorganic nitrogen composition, and aquatic ecosystems stability (Shiozaki et al., 2019) in oceans (Horak et al., 2018; Peng et al., 2018; Shiozaki et al., 2019), rivers (Lipschultz et al., 1985; Merbt et al., 2017), and fishponds (Wu et al., 2020). Here, we demonstrate that comammox *N. inopinata* is also sensitive to light in pure culture with limited tolerance to short dim light exposures (Figures 2A–C; Supplementary Figures S1 and S2). The delayed recovery of *N. inopinata* nitrification activity relative to illumination intensity and duration appears to be comparable with previously observed responses of AOA to light exposure (Merbt et al., 2012; Qin et al., 2014). Photoinhibition of the soil AOA *N. viennensis* in pure culture has not previously been investigated and was included in this study for that reason and to compare the photoinhibition of AOA *N. viennensis* and comammox *N. inopinata*. *N. viennensis* ammonia oxidation appeared to be more sensitive to light exposure than that in *N. inopinata* (Figure 2D). Light sensitivity of the second metabolic step, oxidation of nitrite to nitrate which is unique to comammox and NOB, has yielded the interesting finding that it is quite sensitive to even low levels of light that would not be expected to cause extensive damage to an enzyme, and which have no effect on the ammonium oxidation step (Supplementary Figure S1). Additionally, we found that the wavelength and light intensity influenced the photoinhibition outcome in comammox *N. inopinata*. When incubating cultures at 60 μE m^−2^ s^−1^ for 15 min, blue and green light with less than 550 nm wavelengths inhibited *N. inopinata* activity, while red light with wavelengths greater than 550 nm had little to no impact (Figure 4). Kim and Park (2021) reported higher sensitivity to blue light for NOB than for AOB in an engineered system (Kim and Park, 2021), and similar observations regarding light wavelengths were previously documented for both AOB and AOA (French et al., 2012). Interestingly, photoinhibition was observed to be species dependent for both AOA (Qin et al., 2014) and AOB (Merbt et al., 2012) with microorganisms demonstrating varying degrees of photosensitivity. We show that comammox *N. inopinata* is highly sensitive to light; however, other comammox species need to be evaluated for their sensitivity to light in order to draw conclusion about photoinhibition driven niche differentiation of comammox in various ecosystems.

Metabolic studies have demonstrated that light damages the copper-containing AMO enzyme in AOB (Hooper and Terry, 1974; Shears and Wood, 1985; Hyman and Arp, 1992; Stein et al., 2000) that was proposed to be photoinactivated during simultaneous ammonia conversion into hydroxylamine (NH_2_OH) and exposure to light (Lu et al., 2020a). *De novo* AMO synthesis is required to restore ammonia oxidation after these bacteria are exposed to light (Guerrero and Jones, 1996a,b; Hyman and Arp, 1992). Consequently, conditions preventing valence state changes in AMO’s copper ions (Shears and Wood, 1985), and therefore preventing AMO activation, such as oxygen and ammonia absence, play a protective role for AMO during light exposure (Hooper and Terry, 1974; Hyman and Arp, 1992; Juliette et al., 1993; Ward, 2013). In contrast, when NH_3_ is present, high NH_3_ substrate concentration can shield AMO from light inactivation in AOB (Hooper and Terry, 1974). Most of the research on AMO photoinactivation mechanisms has been performed on AOB organisms leaving the AMO photoinactivation processes in AOA largely uncharacterized. Genome studies of AOA have shown their genomes contain a large number of copper-containing enzymes such as AMO, putative copper-containing nitrite reductase, plastocyanin, and other multicopper oxidases and blue copper proteins (Walker et al., 2010; Blainey et al., 2011), suggesting that some other copper-containing enzymes in AOA could also be sensitive to light, possibly leading to inhibition of ammonia oxidation and/or electron transfer in AOA by light. Comammox *amo* genes are phylogenetically distinct from AOA and AOB *amo* subunits; however, they are homologous to the AOB/AOA *amo* genes (Daims et al., 2015). AMO proteins in comammox are likely inhibited by light *via* a similar mechanism as in AOB and AOA. However, more detailed studies focusing on mechanistic aspects of AMO photoinactivation and the inactivation of other enzymes involved in nitrification in comammox are needed to draw stronger conclusions.

Nitrite-oxidizing activity of NOB species in both natural systems and laboratory settings has been shown to be affected by differential light exposures (Mueller-Neuglueck and Engel, 1961; Bock, 1965; Olson, 1981; Yoshioka and Saijo, 1984; Diab and Shilo, 1988; Vanzella et al., 1989; Guerrero and Jones, 1996a,b; Kaplan et al., 2000; Marks et al., 2012). Canonical *Nitrospira* were shown to dominate ecosystems when kept under dark conditions (Marks et al., 2012). Several studies demonstrated that photoinhibition of nitrite oxidation was more pronounced than ammonia oxidation (Yoshioka and Saijo, 1984; Diab and Shilo, 1988; Vergara et al., 2016); however in some instances, duration of illumination and substrate availability influenced the outcome of photoinhibition on AOB and NOB representatives (Yoshioka and Saijo, 1984). Other reports indicate that nitrite oxidation recovery after photoinhibition took longer than ammonia oxidation recovery when cultures were placed back into dark conditions (Guerrero and Jones, 1996a,b). Additionally, NOB species were found to be more sensitive than AOB to blue light irradiation (Guerrero and Jones, 1996a,b; Guerrero and Jones, 1997; Kim and Park, 2021). In wastewater treatment bioreactors, light irradiation showed higher inhibition of the NOB (*Nitrospira*) than AOB through inducing different transcriptional responses, as *nxrB* gene expression was largely suppressed while the *amoA* expression was less affected by light radiation (Wang et al., 2021a). While several studies compare photoinhibition effect on AOM and NOB, there are a lot of variations in the literature in physiological responses of nitrifiers to abiotic factors due to limited cultured representative of AOA, AOB, and NOB and even more sparse pure cultures availability, differences in studies design, and populations complexities (for example, field analysis vs. using laboratory analysis) making it difficult to draw a comprehensive picture of environmental factors influencing nitrification activity among nitrifiers. We observed that during diurnal light regimen, comammox *N. inopinata* demonstrates delayed and surprisingly interrupted complete nitrification activity demonstrating nitrite accumulation and leading to therefore ceased nitrate production (Figure 5A). This unexpected result suggests that the nitrite oxidation step of comammox metabolism is more susceptible to repeated prolonged illumination than ammonia oxidation. Observed in this study delay in nitrification activity after 60 min of dim light exposure (Figs. 2A, B) may explain why nitrate production is stopped during diurnal light exposure as cultures do not have sufficient time to recover during 16 h of dark regimen before the next round of illumination happens. This suggests that a gene regulation may be involved in the recovery of ammonia oxidation; however, more experiments are necessary to explore specifically the molecular responses of comammox to light.

The widespread distribution of comammox in natural and man-made environments and its important role in the nitrogen cycle highlights the importance of ecophysiology studies to better understand its spatial localization within microbial communities. Abiotic factors that have been presently identified to strongly influence abundance and structure of comammox *Nitrospira* include pH, salinity, iron availability, total oxygen content, ammonia and nitrite availability, and sediment particle size (Sun et al., 2020). Salinity was proposed to play a crucial role in comammox distribution preventing its ocean colonization (Kuypers, 2017; Santos et al., 2018) and showed a negative effect on comammox (Sun et al., 2020). Photoinhibition has been thought to play a role in the spatial distribution of AOM in water columns and for the restriction of the nitrification to the low light levels present at the bottom of the euphotic zone and deeper in aquatic systems (French et al., 2012; Merbt et al., 2017; Horak et al., 2018). Light exposure is an important abiotic factor for many microorganisms, but the effect of light on the distribution of comammox species remains poorly understood. While comammox species so far have not been found in open oceans where photoinhibition effect was evaluated for other AOMs, comammox is prevalent in many other aquatic habitats that experience light irradiation (Liu et al., 2020; Sun et al., 2020). One such area is riparian zones where comammox abundance fluctuates depending on the wet/dry seasons: comammox was present mostly in water during wet season and in sediment during dry season (Lu et al., 2020b). Riparian zones are the areas in nature where ammonia removal occurs and where oxic/anoxic zones can be found (Naiman and Décamps, 1997; McClain et al., 2003). Comammox is proposed to have better survival chances in nutrient-poor environment due to its high ammonia affinity and metabolic versatility (Daims et al., 2015; Fowler et al., 2018; Koch et al., 2019). Comammox consequently was found to be located in fringe soils between rhizosphere and non-rhizosphere in riparian zones where microaerobic conditions and low ammonia concentrations are found (Wang et al., 2021b). Besides providing a favorable environment for comammox, riparian forests also provide shaded conditions (Yoshimura and Kubota, 2022) that can protect comammox from light overexposure. Ecologically significant interactions between abiotic factors and AOM are complex and not fully understood, but it is clear that many AOM are found in normally dark locations and are quite sensitive to light so this remains a factor that must be considered. Evaluating comammox spatial distribution with respect to light penetration in aquatic habitats may help to evaluate the importance of light inhibition in controlling the distribution and activity of these AOM populations that now thought to play an essential role in shaping the nitrogen cycle in freshwater ecosystems.

Photochemically produced reactive oxygen species (ROS) including hydrogen peroxide (H_2_O_2_) arise from photooxidation of chromophoric dissolved organic matter (Cooper et al., 1994) and can indirectly contribute to photoinhibition (Morris et al., 2011; Baltar et al., 2013). H_2_O_2_ has been shown to negatively affect nitrification rates in the ocean with region and taxa specificity (Luo et al., 2014; Tolar et al., 2016). Although this inhibition effect of H_2_O_2_ can be taxa specific, nitrification rates of some AOB and AOA species were not affected by H_2_O_2_ in the absence of peroxidase or catalase genes (Walker et al., 2010; Qin et al., 2017; Horak et al., 2018). Addition of H_2_O_2_ scavengers such as α-Keto acids helps to alleviate the negative effect of light on AOA (Kim et al., 2016). While the elevated AOA nitrification rates upon addition of α-Keto acid have been attributed to mixotrophy, pyruvate incorporation into archaeal lipid membranes was insignificant (Kim et al., 2016). Instead, α-Keto acids spontaneously detoxify H_2_O_2_ using suggested nonenzymatic decarboxylation reaction in a similar way as dimethylthiourea and catalase perform their H_2_O_2_ inactivation (Kim et al., 2016). In this study, we tested the effect of pyruvate, an α-Keto acid, as supplementation on *N. inopinata* comammox reaction under differential light intensities and found that at higher light intensities (60 and 500 μE m^−2^ S^−1^) pyruvate appears to play protective role against photoinhibition (Figure 4). Although comammox *N. inopinata* is a catalase-positive species (Daims et al., 2015), capable of withstanding oxidative stress from H_2_O_2_ accumulation (RedCorn et al., 2022), the supplementation of pyruvate may facilitate the removal of ROS under high light exposure. Further studies are needed to explore the role of pyruvate in comammox *N. inopinata* physiology, especially under light irradiation. Additionally, it is necessary to evaluate ROS production by *N. inopinata* during light exposure.

Taken together, our study provides evidence that comammox *N. inopinata* is inhibited by light at low intensities, but it can tolerate short intermittent light exposures. We also found that similarly to other AOM comammox *N. inopinata* nitrification activity is more susceptible to light with shorter wavelengths. The degree of *N. inopinata* photoinhibition is similar to AOA species yet *N. inopinata* appears to be more sensitive than some AOA species to light exposure and exhibits slower or non-existent recovery after being placed back in the dark. However, photoinhibition of the soil AOA *N. viennensis* appears to be even stronger than in *N. inopinata.* Light likely inactivates AMO enzyme in *N. inopinata* and may potentially damage other enzymes involved in the second step of nitrification as suggested by the ceased nitrite oxidation of comammox under the diurnal light exposure. Differences in comammox metabolisms from other AOM may also reflect possible unique modes of photoinhibition in comammox and strategies to cope with the light-damaging effect. It would be interesting to compare latitudinal variations in comammox distribution in similar physiochemical environments that differ in diurnal regimens.

## Data availability statement

The raw data supporting the conclusions of this article will be made available by the authors, without undue reservation.

## Author contributions

EG and BG contributed to conception and design of the study, data analysis, and manuscript revision. EG wrote the first draft of the manuscript. BL, BA, and WQ contributed to manuscript revision. MW contributed to conception and design of the study and manuscript revisions. All authors read and approved the submitted version. All authors contributed to the article and approved the submitted version.

## Funding

EG, BG, BA, and MW received financial support from the Defense Advanced Research Projects Agency [Contract Number: HR0011-17-2-0064] and BG, BA, and MW were also supported by the U.S. Department of Energy (DOE) [Contract Number: DE-SC0020356]. BL was supported by Washington Research Foundation Project 5117.

## Conflict of interest

The authors declare that the research was conducted in the absence of any commercial or financial relationships that could be construed as a potential conflict of interest.

## Publisher’s note

All claims expressed in this article are solely those of the authors and do not necessarily represent those of their affiliated organizations, or those of the publisher, the editors and the reviewers. Any product that may be evaluated in this article, or claim that may be made by its manufacturer, is not guaranteed or endorsed by the publisher.

## References

[ref1] Auguet J.-C. Casamayor E. O. (2013). Partitioning of Thaumarchaeota populations along environmental gradients in high mountain lakes. FEMS Microbiol. Ecol. 84, 154–164. doi: 10.1111/1574-6941.12047, PMID: 23176712

[ref2] Auguet J.-C. Triadó-Margarit X. Nomokonova N. Camarero L. Casamayor E. O. (2012). Vertical segregation and phylogenetic characterization of ammonia-oxidizing archaea in a deep oligotrophic lake. ISME J. 6, 1786–1797. doi: 10.1038/ismej.2012.33, PMID: 22495069 PMC3425235

[ref3] Baltar F. Reinthaler T. Herndl G. J. Pinhassi J. (2013). Major effect of hydrogen peroxide on Bacterioplankton metabolism in the Northeast Atlantic. PLoS One 8:e61051. doi: 10.1371/journal.pone.0061051, PMID: 23593386 PMC3625187

[ref4] Bartelme R. P. McLellan S. L. Newton R. J. (2017). Freshwater recirculating Aquaculture system operations drive biofilter bacterial community shifts around a stable nitrifying consortium of ammonia-oxidizing archaea and comammox *Nitrospira*. Front. Microbiol. 8:101. doi: 10.3389/fmicb.2017.00101, PMID: 28194147 PMC5276851

[ref5] Bernhard A. E. Bollmann A. (2010). Estuarine nitrifiers: new players, patterns and processes. Estuar. Coast. Shelf Sci. 88, 1–11. doi: 10.1016/j.ecss.2010.01.023

[ref6] Blainey P. C. Mosier A. C. Potanina A. Francis C. A. Quake S. R. (2011). Genome of a low-salinity ammonia-oxidizing archaeon determined by single-cell and metagenomic analysis. PLoS One 6:e16626. doi: 10.1371/journal.pone.0016626, PMID: 21364937 PMC3043068

[ref7] Bock E. (1965). Comparative studies on the effect of visible light on Nitrosomonas europaea and Nitrobacter winogradsky. Arch. Mikrobiol. 51, 18–41. doi: 10.1007/BF00406848 14347921

[ref8] Bollmann A. French E. Laanbroek H. J. (2011). “Chapter three - isolation, cultivation, and characterization of ammonia-oxidizing bacteria and archaea adapted to low ammonium concentrations” in Methods in Enzymology. ed. Klotz M. G. (Cambridge: Academic Press), 55–88. doi: 10.1016/B978-0-12-381294-0.00003-1 21185431

[ref9] Camejo P. Y. Santo Domingo J. McMahon K. D. Noguera D. R. (2017). Genome-enabled insights into the ecophysiology of the comammox bacterium Candidatus *Nitrospira nitrosa*. mSystems 2, e00059–e00017. doi: 10.1128/mSystems.00059-17 28905001 PMC5596200

[ref10] Cooper W.J. Shao C. Lean D.R.S. Gordon A.S. Scully F.E. (1994). “Factors affecting the distribution of H_2_O_2_ in surface waters,” in Environmental Chemistry of Lakes and Reservoirs, Advances in Chemistry. ed. L. A. Baker. New York: American Chemical Society, pp. 391–422.

[ref11] Daims H. Lebedeva E. V. Pjevac P. Han P. Herbold C. Albertsen M. (2015). Complete nitrification by *Nitrospira* bacteria. Nature 528, 504–509. doi: 10.1038/nature16461, PMID: 26610024 PMC5152751

[ref12] Diab S. Shilo M. (1988). Effect of light on the activity and survival of *Nitrosomonas* sp. and *Nitrobacter* sp. isolates from fish ponds. Israeli J. Aquac. Bamidgeh 40, 50–56.

[ref13] Fowler S. J. Palomo A. Dechesne A. Mines P. D. Smets B. F. (2018). Comammox *Nitrospira* are abundant ammonia oxidizers in diverse groundwater-fed rapid sand filter communities. Environ. Microbiol. 20, 1002–1015. doi: 10.1111/1462-2920.14033, PMID: 29314644

[ref14] French E. Kozlowski J. A. Mukherjee M. Bullerjahn G. Bollmann A. (2012). Ecophysiological characterization of ammonia-oxidizing archaea and bacteria from freshwater. Appl. Environ. Microbiol. 78, 5773–5780. doi: 10.1128/AEM.00432-12, PMID: 22685142 PMC3406153

[ref15] Gonzalez-Martinez A. Rodriguez-Sanchez A. van Loosdrecht M. C. M. Gonzalez-Lopez J. Vahala R. (2016). Detection of comammox bacteria in full-scale wastewater treatment bioreactors using tag-454-pyrosequencing. Environ. Sci. Pollut. Res. 23, 25501–25511. doi: 10.1007/s11356-016-7914-4, PMID: 27783252

[ref16] Guerrero M. A. Jones R. D. (1996a). Photoinhibition of marine nitrifying bacteria. II. Dark recovery after monochromatic or polychromatic irradiation. Mar. Ecol. Prog. Ser. 141, 193–198. doi: 10.3354/meps141193

[ref17] Guerrero M. A. Jones R. D. (1996b). Photoinhibition of marine nitrifying bacteria. I. Wavelength-dependent response. Mar. Ecol. Prog. Ser. 141, 183–192. doi: 10.3354/meps141183

[ref18] Guerrero M. A. Jones R. D. (1997). Light-induced absorbance changes associated with photoinhibition and pigments in nitrifying bacteria. Aquat. Microb. Ecol. 13, 233–239. doi: 10.3354/ame013233

[ref19] He S. Zhao Z. Tian Z. Xu C. Liu Y. He D. (2022). Comammox bacteria predominate among ammonia-oxidizing microorganisms in municipal but not in refinery wastewater treatment plants. J. Environ. Manag. 316:115271. doi: 10.1016/j.jenvman.2022.115271, PMID: 35594823

[ref20] Hooper A. Terry K. (1974). Photoinactivation of ammonia oxidation in Nitrosomonas. J. Bacteriol. 119, 899–906. doi: 10.1128/JB.119.3.899-906.1974, PMID: 4369012 PMC245697

[ref21] Horak R. E. A. Qin W. Bertagnolli A. D. Nelson A. Heal K. R. Han H. (2018). Relative impacts of light, temperature, and reactive oxygen on thaumarchaeal ammonia oxidation in the North Pacific Ocean. Limnol. Oceanogr. 63, 741–757. doi: 10.1002/lno.10665

[ref22] How S. W. Chua A. S. M. Ngoh G. C. Nittami T. Curtis T. P. (2019). Enhanced nitrogen removal in an anoxic-oxic-anoxic process treating low COD/N tropical wastewater: low-dissolved oxygen nitrification and utilization of slowly-biodegradable COD for denitrification. Sci. Total Environ. 693:133526. doi: 10.1016/j.scitotenv.2019.07.332, PMID: 31376760

[ref23] Hu H.-W. He J.-Z. (2017). Comammox—a newly discovered nitrification process in the terrestrial nitrogen cycle. J. Soils Sediments 17, 2709–2717. doi: 10.1007/s11368-017-1851-9

[ref24] Hyman M. R. Arp D. J. (1992). 14C2H2-and 14CO2-labeling studies of the de novo synthesis of polypeptides by Nitrosomonas europaea during recovery from acetylene and light inactivation of ammonia monooxygenase. J. Biol. Chem. 267, 1534–1545. doi: 10.1016/S0021-9258(18)45979-0, PMID: 1730700

[ref25] Juliette L. Y. Hyman M. R. Arp D. J. (1993). Inhibition of ammonia oxidation in Nitrosomonas europaea by sulfur compounds: Thioethers are oxidized to sulfoxides by ammonia monooxygenase. Appl. Environ. Microbiol. 59, 3718–3727. doi: 10.1128/aem.59.11.3718-3727.1993, PMID: 16349086 PMC182523

[ref26] Jung M.-Y. Sedlacek C. J. Kits K. D. Mueller A. J. Rhee S.-K. Hink L. (2022). Ammonia-oxidizing archaea possess a wide range of cellular ammonia affinities. ISME J. 16, 272–283. doi: 10.1038/s41396-021-01064-z, PMID: 34316016 PMC8692354

[ref27] Kaplan D. Wilhelm R. Abeliovich A. (2000). Interdependent environmental factors controlling nitrification in waters. Water Sci. Technol. 42, 167–172. doi: 10.2166/wst.2000.0309

[ref28] Kim K. Park Y.-G. (2021). Light as a novel inhibitor of nitrite-oxidizing bacteria (NOB) for the mainstream partial nitrification of wastewater treatment. PRO 9:346. doi: 10.3390/pr9020346

[ref29] Kim J.-G. Park S.-J. Sinninghe Damsté J. S. Schouten S. Rijpstra W. I. C. Jung M.-Y. (2016). Hydrogen peroxide detoxification is a key mechanism for growth of ammonia-oxidizing archaea. Proc. Natl. Acad. Sci. 113, 7888–7893. doi: 10.1073/pnas.1605501113, PMID: 27339136 PMC4948306

[ref30] Kits K. D. Sedlacek C. J. Lebedeva E. V. Han P. Bulaev A. Pjevac P. (2017). Kinetic analysis of a complete nitrifier reveals an oligotrophic lifestyle. Nature 549, 269–272. doi: 10.1038/nature23679, PMID: 28847001 PMC5600814

[ref31] Koch H. van Kessel M. A. H. J. Lücker S. (2019). Complete nitrification: insights into the ecophysiology of comammox *Nitrospira*. Appl. Microbiol. Biotechnol. 103, 177–189. doi: 10.1007/s00253-018-9486-3, PMID: 30415428 PMC6311188

[ref32] Könneke M. Bernhard A. E. de la Torre J. R. Walker C. B. Waterbury J. B. Stahl D. A. (2005). Isolation of an autotrophic ammonia-oxidizing marine archaeon. Nature 437, 543–546. doi: 10.1038/nature03911, PMID: 16177789

[ref33] Kuypers M. M. M. (2017). Microbiology: a fight for scraps of ammonia. Nature 549, 162–163. doi: 10.1038/549162a, PMID: 28905910

[ref34] Leininger S. Urich T. Schloter M. Schwark L. Qi J. Nicol G. W. (2006). Archaea predominate among ammonia-oxidizing prokaryotes in soils. Nature 442, 806–809. doi: 10.1038/nature04983, PMID: 16915287

[ref35] Lipschultz F. Wofsy S. C. Fox L. E. (1985). The effects of light and nutrients on rates of ammonium transformation in a eutrophic river. Mar. Chem. 16, 329–341. doi: 10.1016/0304-4203(85)90054-4

[ref36] Liu S. Wang H. Chen L. Wang J. Zheng M. Liu S. (2020). Comammox *Nitrospira* within the Yangtze River continuum: community, biogeography, and ecological drivers. ISME J. 14, 2488–2504. doi: 10.1038/s41396-020-0701-8, PMID: 32555502 PMC7490378

[ref37] Lu S. Liu X. Liu C. Cheng G. Shen H. (2020a). Influence of photoinhibition on nitrification by ammonia-oxidizing microorganisms in aquatic ecosystems. Rev. Environ. Sci. Biotechnol. 19, 531–542. doi: 10.1007/s11157-020-09540-2

[ref38] Lu S. Sun Y. Lu B. Zheng D. Xu S. (2020b). Change of abundance and correlation of Nitrospira inopinata-like comammox and populations in nitrogen cycle during different seasons. Chemosphere 241:125098. doi: 10.1016/j.chemosphere.2019.125098, PMID: 31877618

[ref39] Luo H. Tolar B. B. Swan B. K. Zhang C. L. Stepanauskas R. Ann Moran M. (2014). Single-cell genomics shedding light on marine Thaumarchaeota diversification. ISME J. 8, 732–736. doi: 10.1038/ismej.2013.202, PMID: 24196320 PMC3930325

[ref40] Marks C. R. Stevenson B. S. Rudd S. Lawson P. A. (2012). Nitrospira-dominated biofilm within a thermal artesian spring: a case for nitrification-driven primary production in a geothermal setting. Geobiology 10, 457–466. doi: 10.1111/j.1472-4669.2012.00335.x, PMID: 22726612

[ref41] Martens-Habbena W. Berube P.M. Urakawa H. Torre J.R. de la Stahl D.A. (2009). Ammonia oxidation kinetics determine niche separation of nitrifying archaea and bacteria. Nature 461, 976–979. doi: 10.1038/nature08465, PMID: 19794413

[ref42] McClain M. E. Boyer E. W. Dent C. L. Gergel S. E. Grimm N. B. Groffman P. M. (2003). Biogeochemical hot spots and hot moments at the Interface of terrestrial and aquatic ecosystems. Ecosystems 6, 301–312. doi: 10.1007/s10021-003-0161-9

[ref43] Merbt S. N. Bernal S. R. Proia L. Martí E. Casamayor E. O. (2017). Photoinhibition on natural ammonia oxidizers biofilm populations and implications for nitrogen uptake in stream biofilms. Limnol. Oceanogr. 62, 364–375. doi: 10.1002/lno.10436

[ref44] Merbt S. N. Stahl D. A. Casamayor E. O. Martí E. Nicol G. W. Prosser J. I. (2012). Differential photoinhibition of bacterial and archaeal ammonia oxidation. FEMS Microbiol. Lett. 327, 41–46. doi: 10.1111/j.1574-6968.2011.02457.x, PMID: 22093004

[ref45] Morris J. J. Johnson Z. I. Szul M. J. Keller M. Zinser E. R. (2011). Dependence of the cyanobacterium Prochlorococcus on hydrogen peroxide scavenging microbes for growth at the Ocean’s surface. PLoS One 6:e16805. doi: 10.1371/journal.pone.0016805, PMID: 21304826 PMC3033426

[ref46] Mueller-Neuglueck M. Engel H. (1961). Photoinactivation of NItrobacter winogradskyi Buch. Arch. Mikrobiol. 39, 130–138. doi: 10.1007/BF00408615 13773574

[ref47] Naiman R. J. Décamps H. (1997). The ecology of interfaces: riparian zones. Annu. Rev. Ecol. Syst. 28, 621–658. doi: 10.1146/annurev.ecolsys.28.1.621

[ref48] Nicol G. W. Leininger S. Schleper C. Prosser J. I. (2008). The influence of soil pH on the diversity, abundance and transcriptional activity of ammonia oxidizing archaea and bacteria. Environ. Microbiol. 10, 2966–2978. doi: 10.1111/j.1462-2920.2008.01701.x, PMID: 18707610

[ref49] Olson R. (1981). Differential photoinhibition of marine nitrifying bacteria: a possible mechanism for the formation of the primary nitrite maximum. J. Mar. Res. 39, 227–238.

[ref50] Orellana L. H. Chee-Sanford J. C. Sanford R. A. Löffler F. E. Konstantinidis K. T. (2018). Year-round shotgun metagenomes reveal stable microbial communities in agricultural soils and novel ammonia oxidizers responding to fertilization. Appl. Environ. Microbiol. 84, e01646–e01617. doi: 10.1128/AEM.01646-17, PMID: 29101194 PMC5752871

[ref51] Peng X. Fawcett S. E. van Oostende N. Wolf M. J. Marconi D. Sigman D. M. (2018). Nitrogen uptake and nitrification in the subarctic North Atlantic Ocean. Limnol. Oceanogr. 63, 1462–1487. doi: 10.1002/lno.10784

[ref52] Pinto A. J. Marcus D. N. Ijaz U. Z. Bautista-de L. S. Q. M. Dick G. J. Raskin L. (2016). Metagenomic evidence for the presence of comammox *Nitrospira*-like bacteria in a drinking water system. mSphere 1, e00054–e00015. doi: 10.1128/mSphere.00054-15 27303675 PMC4863621

[ref53] Pjevac P. Schauberger C. Poghosyan L. Herbold C. W. van Kessel M. A. H. J. Daebeler A. (2017). Amo A-targeted polymerase chain reaction primers for the specific detection and quantification of comammox *Nitrospira* in the environment. Front. Microbiol. 8:1508. doi: 10.3389/fmicb.2017.01508, PMID: 28824606 PMC5543084

[ref54] Qin W. Amin S. A. Martens-Habbena W. Walker C. B. Urakawa H. Devol A. H. (2014). Marine ammonia-oxidizing archaeal isolates display obligate mixotrophy and wide ecotypic variation. Proc. Natl. Acad. Sci. U. S. A. 111, 12504–12509. doi: 10.1073/pnas.1324115111, PMID: 25114236 PMC4151751

[ref55] Qin W. Meinhardt K. A. Moffett J. W. Devol A. H. Virginia Armbrust E. Ingalls A. E. (2017). Influence of oxygen availability on the activities of ammonia-oxidizing archaea. Environ. Microbiol. Rep. 9, 250–256. doi: 10.1111/1758-2229.12525, PMID: 28211189

[ref56] RedCorn R. Lamb J. R. Gottshall E. Stahl D. A. Winkler M. K. H. (2022). Light-weight oxygen supply for portable biological nitrogen removal from urine and sweat. Chem. Eng. J. Adv. 9:100235. doi: 10.1016/j.ceja.2021.100235

[ref57] Roland H. (2012). Diversity, physiology, and niche differentiation of ammonia-oxidizing archaea. Appl. Environ. Microbiol. 78, 7501–7510. doi: 10.1128/AEM.01960-12, PMID: 22923400 PMC3485721

[ref58] Roots P. Wang Y. Rosenthal A. F. Griffin J. S. Sabba F. Petrovich M. (2019). Comammox *Nitrospira* are the dominant ammonia oxidizers in a mainstream low dissolved oxygen nitrification reactor. Water Res. 157, 396–405. doi: 10.1016/j.watres.2019.03.060, PMID: 30974288

[ref59] Santos J. P. Mendes D. Monteiro M. Ribeiro H. Baptista M. S. Borges M. T. (2018). Salinity impact on ammonia oxidizers activity and amo a expression in estuarine sediments. Estuar. Coast. Shelf Sci. 211, 177–187. doi: 10.1016/j.ecss.2017.09.001

[ref60] Schoen G. H. Engel H. (1962). The effect of light on Nitrosomonas europaea win. Arch. Mikrobiol. 42, 415–428. doi: 10.1007/BF00409076 13909041

[ref61] Shears J. Wood P. (1985). Spectroscopic evidence for a photosensitive oxygenated state of ammonia mono-oxygenase. Biochem. J. 226, 499–507. doi: 10.1042/bj2260499, PMID: 3922353 PMC1144737

[ref62] Shiozaki T. Ijichi M. Fujiwara A. Makabe A. Nishino S. Yoshikawa C. (2019). Factors regulating nitrification in the Arctic Ocean: potential impact of sea ice reduction and ocean acidification. Glob. Biogeochem. Cycles 33, 1085–1099. doi: 10.1029/2018GB006068

[ref63] Spasov E. Tsuji J. M. Hug L. A. Doxey A. C. Sauder L. A. Parker W. J. (2020). High functional diversity among *Nitrospira* populations that dominate rotating biological contactor microbial communities in a municipal wastewater treatment plant. ISME J. 14, 1857–1872. doi: 10.1038/s41396-020-0650-2, PMID: 32332864 PMC7305129

[ref64] Stein L. Y. Sayavedra-Soto L. A. Hommes N. G. Arp D. J. (2000). Differential regulation of amoA and amoB gene copies in Nitrosomonas europaea. FEMS Microbiol. Lett. 192, 163–168. doi: 10.1111/j.1574-6968.2000.tb09376.x, PMID: 11064189

[ref65] Straka L. L. Meinhardt K. A. Bollmann A. Stahl D. A. Winkler M.-K. H. (2019). Affinity informs environmental cooperation between ammonia-oxidizing archaea (AOA) and anaerobic ammonia-oxidizing (Anammox) bacteria. ISME J. 13, 1997–2004. doi: 10.1038/s41396-019-0408-x, PMID: 30936420 PMC6775968

[ref66] Sun D. Tang X. Zhao M. Zhang Z. Hou L. Liu M. (2020). Distribution and diversity of comammox *Nitrospira* in coastal wetlands of China. Front. Microbiol. 11:2480. doi: 10.3389/fmicb.2020.589268, PMID: 33123118 PMC7573150

[ref67] Tolar B. B. Powers L. C. Miller W. L. Wallsgrove N. J. Popp B. N. Hollibaugh J. T. (2016). Ammonia oxidation in the ocean can be inhibited by Nanomolar concentrations of hydrogen peroxide. Front. Mar. Sci. 3:237.

[ref68] Tourna M. Freitag T. E. Nicol G. W. Prosser J. I. (2008). Growth, activity and temperature responses of ammonia-oxidizing archaea and bacteria in soil microcosms. Environ. Microbiol. 10, 1357–1364. doi: 10.1111/j.1462-2920.2007.01563.x, PMID: 18325029

[ref69] Tourna M. Stieglmeier M. Spang A. Könneke M. Schintlmeister A. Urich T. (2011). Nitrososphaera viennensis, an ammonia oxidizing archaeon from soil. Proc. Natl. Acad. Sci. U. S. A. 108, 8420–8425. doi: 10.1073/pnas.1013488108, PMID: 21525411 PMC3100973

[ref70] van Kessel M. A. H. J. Speth D. R. Albertsen M. Nielsen P. H. Op den Camp H. J. M. Kartal B. (2015). Complete nitrification by a single microorganism. Nature 528, 555–559. doi: 10.1038/nature16459, PMID: 26610025 PMC4878690

[ref71] Vanzella A. Guerrero M. A. Jones R. D. (1989). Effect of CO and light on ammonium and nitrite oxidation by chemolithotrophic bacteria. Mar. Ecol. Prog. Ser. 57, 69–76. doi: 10.3354/meps057069

[ref72] Vergara C. Muñoz R. Campos J. L. Seeger M. Jeison D. (2016). Influence of light intensity on bacterial nitrifying activity in algal-bacterial photobioreactors and its implications for microalgae-based wastewater treatment. Int. Biodeterior. Biodegradation 114, 116–121. doi: 10.1016/j.ibiod.2016.06.006

[ref73] Verhamme D. T. Prosser J. I. Nicol G. W. (2011). Ammonia concentration determines differential growth of ammonia-oxidising archaea and bacteria in soil microcosms. ISME J. 5, 1067–1071. doi: 10.1038/ismej.2010.191, PMID: 21228892 PMC3131854

[ref74] Walker C. B. de la Torre J. R. Klotz M. G. Urakawa H. Pinel N. Arp D. J. (2010). Nitrosopumilus maritimus genome reveals unique mechanisms for nitrification and autotrophy in globally distributed marine crenarchaea. Proc. Natl. Acad. Sci. U. S. A. 107, 8818–8823. doi: 10.1073/pnas.0913533107, PMID: 20421470 PMC2889351

[ref75] Wang L. Qiu S. Guo J. Ge S. (2021a). Light irradiation enables rapid start-up of Nitritation through suppressing nxrB gene expression and stimulating ammonia-oxidizing bacteria. Environ. Sci. Technol. 55, 13297–13305. doi: 10.1021/acs.est.1c04174, PMID: 34529402

[ref76] Wang D.-Q. Zhou C.-H. Nie M. Gu J.-D. Quan Z.-X. (2021b). Abundance and niche specificity of different types of complete ammonia oxidizers (comammox) in salt marshes covered by different plants. Sci. Total Environ. 768:144993. doi: 10.1016/j.scitotenv.2021.144993, PMID: 33736320

[ref77] Ward B. B. (2013). “Nitrification☆” in Encyclopedia of Ecology. ed. Fath B.. 2nd ed (Oxford: Elsevier), 351–358. doi: 10.1016/B978-0-12-409548-9.00697-7

[ref78] Wu D. Cheng M. Zhao S. Peng N. Hu R. Hu J. (2020). Algal growth enhances light-mediated limitation of bacterial nitrification in an Aquaculture system. Water Air Soil Pollut. 231:73. doi: 10.1007/s11270-020-4436-y

[ref79] Wuchter C. Abbas B. Coolen M.J.L. Herfort L. Bleijswijk J. van Timmers P. (2006). Archaeal nitrification in the ocean. PNAS 103, 12317–12322. doi: 10.1073/pnas.0600756103, PMID: 16894176 PMC1533803

[ref80] Xia F. Wang J.-G. Zhu T. Zou B. Rhee S.-K. Quan Z.-X. (2018). Ubiquity and diversity of complete ammonia oxidizers (comammox). Appl. Environ. Microbiol. 84:e01390-18. doi: 10.1128/AEM.01390-18, PMID: 30315079 PMC6275355

[ref81] Yang Y. Daims H. Liu Y. Herbold C. W. Pjevac P. Lin J.-G. (2020). Activity and metabolic versatility of complete ammonia oxidizers in full-scale wastewater treatment systems. MBio 11, 11, e03175–e03119. doi: 10.1128/mBio.03175-19, PMID: 32184251 PMC7078480

[ref82] Yoshimura M. Kubota T. (2022). Evaluation of sunlight penetration through riparian forest and its effects on stream biota. Glob. Ecol. Conserv. 34:e02043. doi: 10.1016/j.gecco.2022.e02043

[ref83] Yoshioka T. Saijo Y. (1984). Photoinhibition and recovery of NH_4_^+^-oxidizing bacteria and NO_2_^−^-oxidizing bacteria. J. Gen. Appl. Microbiol. 30, 151–166. doi: 10.2323/jgam.30.151

[ref84] Zhao J. Bello M. O. Meng Y. Prosser J. I. Gubry-Rangin C. (2020). Selective inhibition of ammonia oxidising archaea by simvastatin stimulates growth of ammonia oxidising bacteria. Soil Biol. Biochem. 141:107673. doi: 10.1016/j.soilbio.2019.107673

