# Supplementary material for: Photoinhibition of comammox reaction in Nitrospira inopinata in a dose- and wavelength-dependent manner
Source: Front Microbiol. 2022 Dec 15;13:1022899. doi: 10.3389/fmicb.2022.1022899 (PMC9797979; doi:10.3389/fmicb.2022.1022899)
Supplement: Supplementary file 1 [file Data_Sheet_1.docx]

**Supplementary Information for**

Photoinhibition of Comammox reaction in *Nitrospira* *inopinata* in a dose and wavelength-dependent manner.

Ekaterina Y Gottshall*^1^, Bruce Godfrey^1^, Bo Li^1^, Britt Abrahamson^1^, Wei Qin^2^, Mari Winkler^1^

^1^ Department of Civil and Environmental Engineering, University of Washington, Seattle, WA, United States.

^2^ Department of Microbiology and Plant Biology, Institute for Environmental Genomics, University of Oklahoma, Norman, OK, 73019

* Corresponding author: [egottshall1@gmail.com](mailto:egottshall1@gmail.com)

Supplementary Figures

| 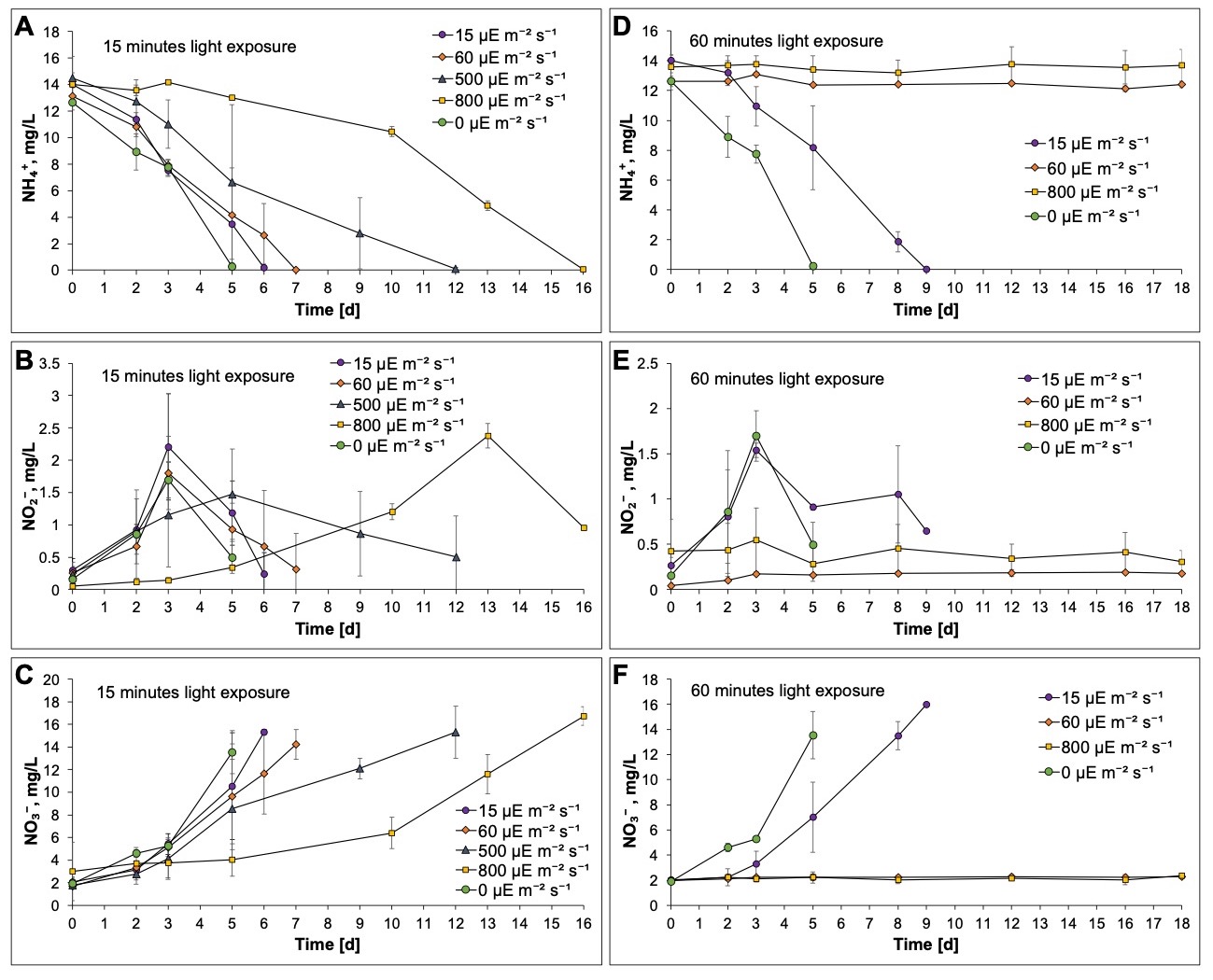 |
| --- |
| **Suppl. Figure 1.** Temporal response of ammonium (**A** and **D**), nitrite (**B** and **E**), and nitrate (**C** and **F**) concentration in *N. inopinata* cultures after exposure to 0, 15, 60, 500, and 800 μE m^−2^ s^−1^ of white light for either 15 minutes (**A-C**) or 60 minutes (**D-F**). Light exposure delayed ammonia and nitrite oxidation activity in *N. inopinata* after exposure to all light intensities for 15 minutes and an intensity of 15 μE m^−2^ s^−1^ for 60 minutes. Nitrification activity in *N. inopinata* ceased after exposure to light intensities of 60 μE m^−2^ s^−1^ or greater for 60 minutes. Data are presented as the mean and standard error of triplicate cultures. |

| 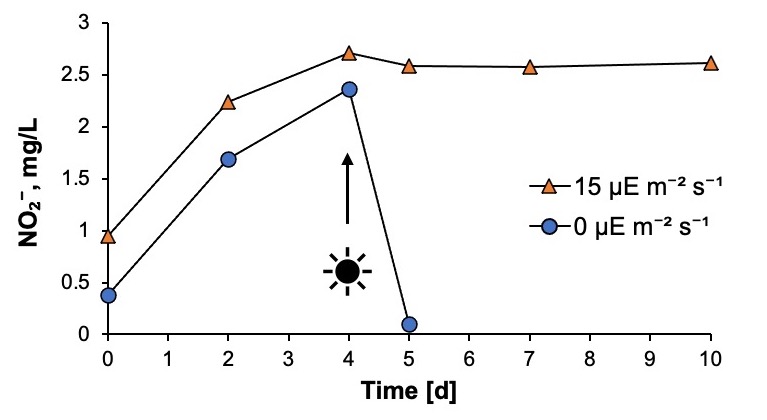 |
| --- |
| **Suppl. Figure 2.** Temporal response in nitrite production/consumption of *N. inopinata* to continuous illumination exposure at an intensity of 0 and 15 μE m^−2^ s^−1^ starting on day 4 of the incubation. The onset of light exposure is indicated by a sun symbol. The continued presence of nitrite in the culture exposed to the 15 μE m^−2^ s^−1^ light intensity shows that continuous low intensity light exposure inactivates nitrite oxidation in *N. inopinata*. |

| 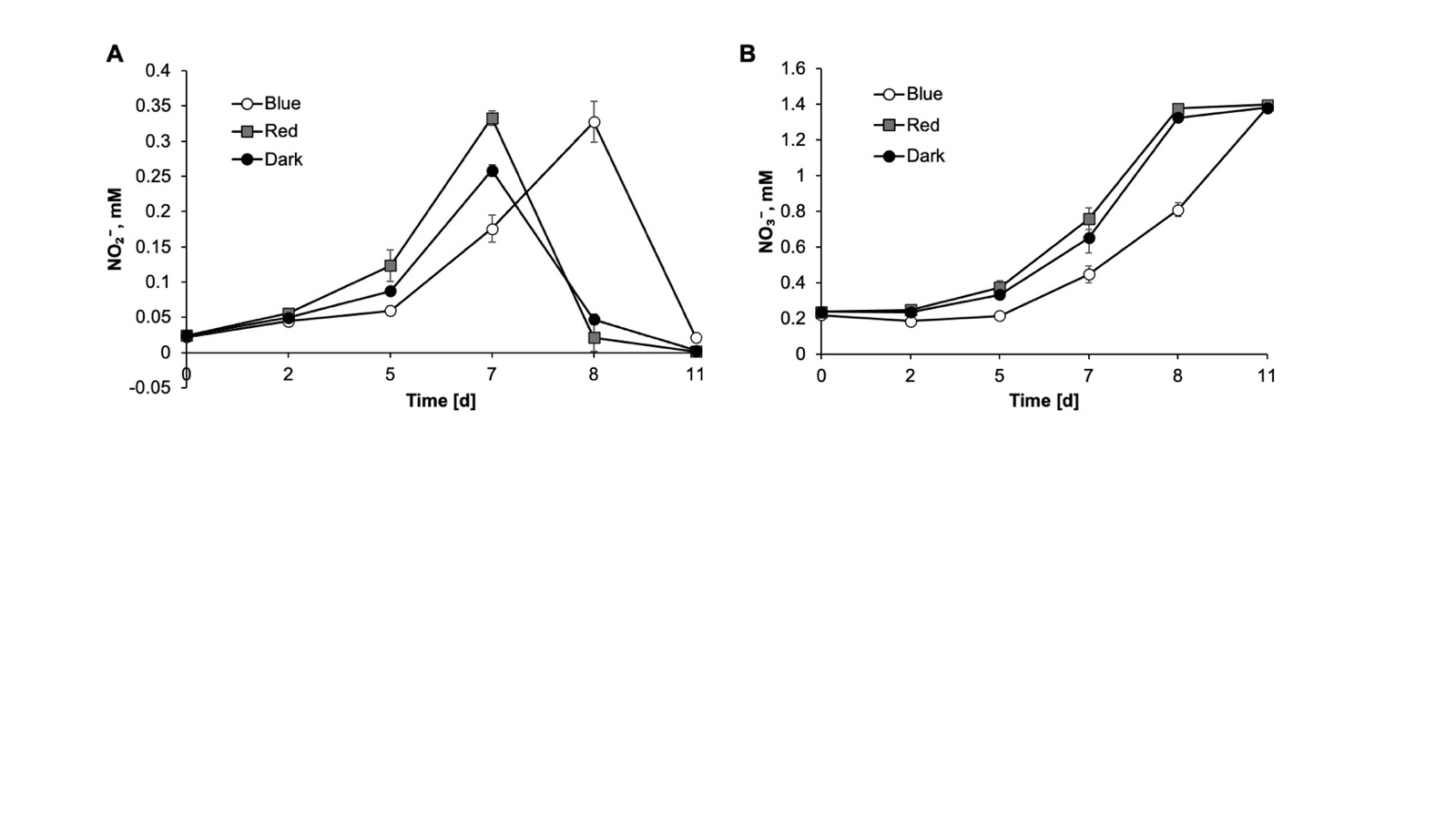 |
| --- |
| **Suppl. Figure 3.** Fifteen minutes exposure of *N. inopinata* to wavelengths of 60 μE m^−2^ s^−1^ blue-green spectrum (450-550 nm) caused delayed nitrite oxidation (**A**) and nitrate production (**B**) in comparison to 60 μE m^−2^ s^−1^ yellow-red spectrum (550-680 nm) wavelengths and dark control. Data are presented as the mean and standard error of triplicate cultures. |

| 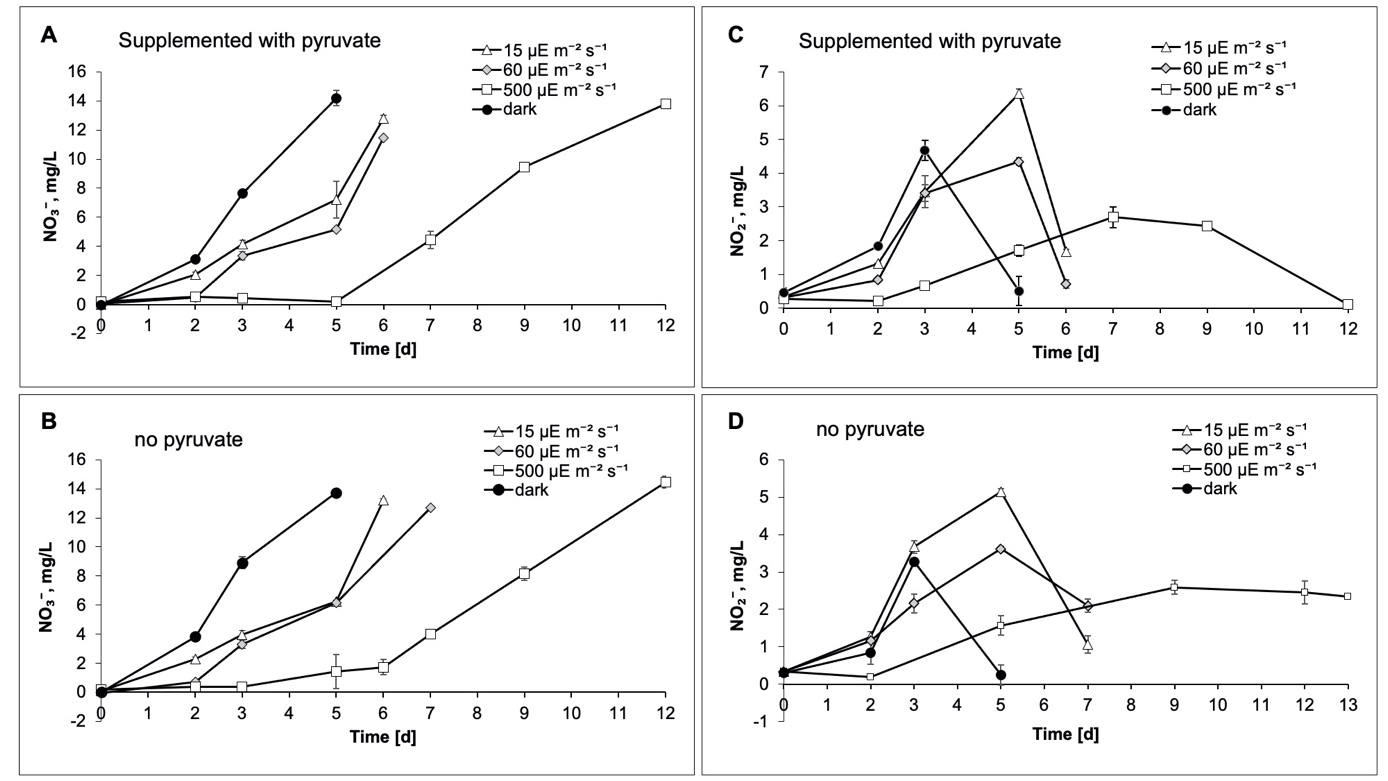 |
| --- |
| **Suppl. Figure 4.** Temporal response of nitrate (**A** and **B**), and nitrite (**C** and **D**) concentration in *N. inopinata* cultures after exposure to 0, 15, 60, and 500 μE m^−2^ s^−1^ of white light for either 15 minutes with or without supplementation with pyruvate. Light exposure delayed nitrification activity in *N. inopinata* after exposure to all light intensities for 15 minutes. Data are presented as the mean and standard error of triplicate cultures. |
