# Supplementary figures and images for: Photoinhibition of comammox reaction in Nitrospira inopinata in a dose- and wavelength-dependent manner
Source: Front Microbiol. 2022 Dec 15;13:1022899. doi: 10.3389/fmicb.2022.1022899 (PMC9797979; doi:10.3389/fmicb.2022.1022899)

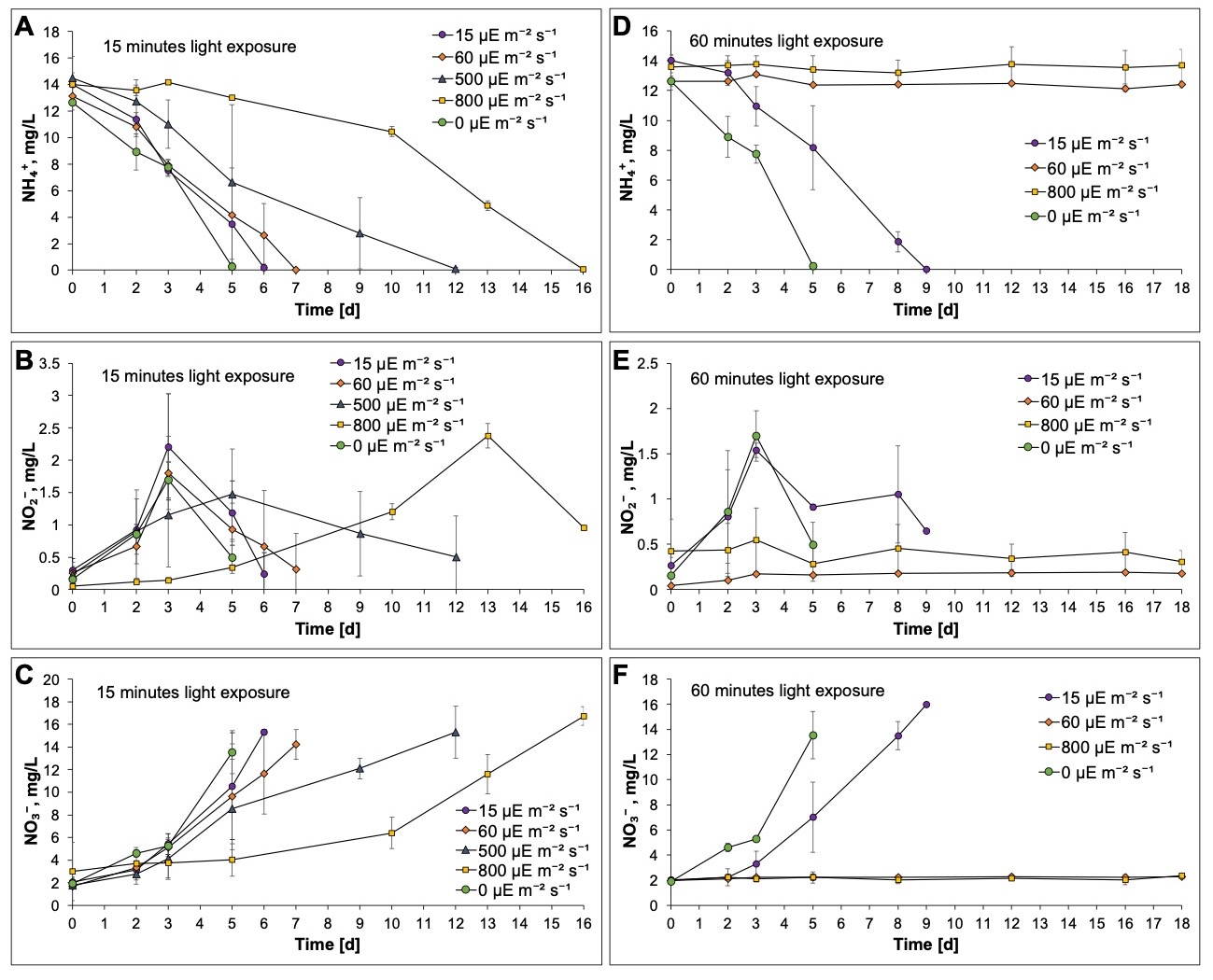

Supplement: Supplementary file 2 [file Image_1.jpg]

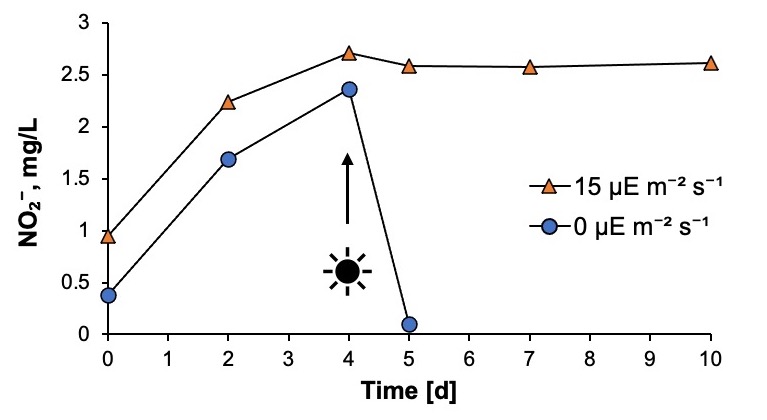

Supplement: Supplementary file 3 [file Image_2.jpg]

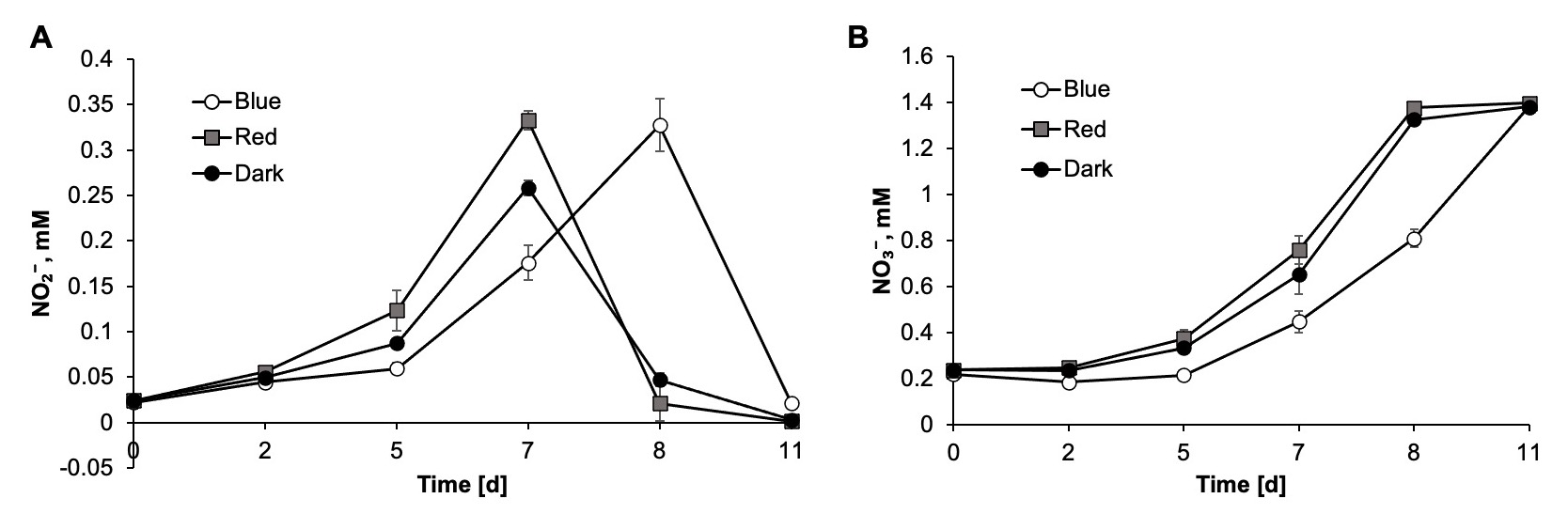

Supplement: Supplementary file 4 [file Image_3.jpg]

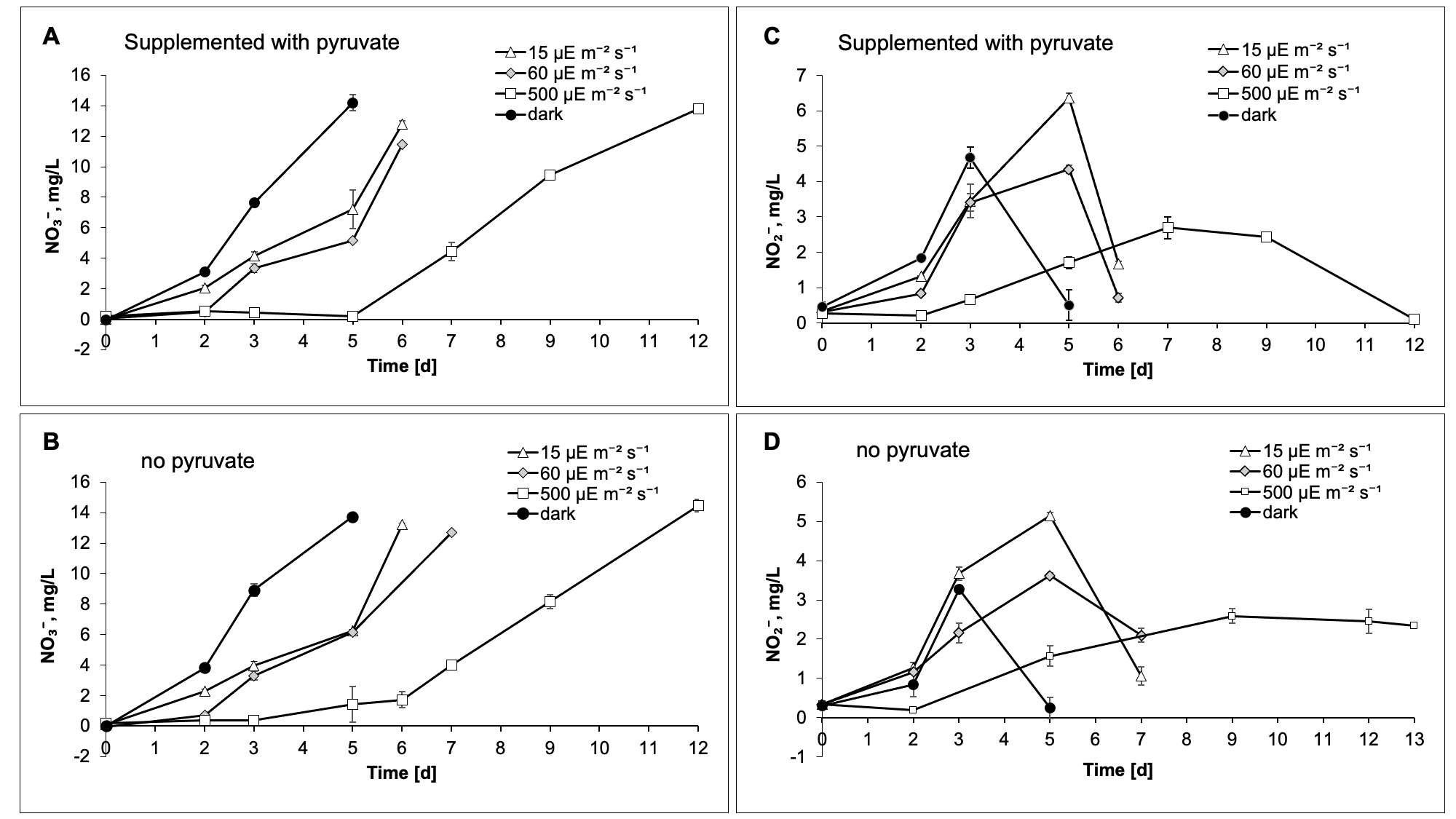

Supplement: Supplementary file 5 [file Image_4.jpg]
